# Input-specific control of interneuron numbers in nascent striatal networks

**DOI:** 10.1073/pnas.2118430119

**Published:** 2022-05-09

**Authors:** Varun Sreenivasan, Eleni Serafeimidou-Pouliou, David Exposito-Alonso, Kinga Bercsenyi, Clémence Bernard, Sung-Eun Bae, Fazal Oozeer, Alicia Hanusz-Godoy, Robert H. Edwards, Oscar Marín

**Affiliations:** ^a^Centre for Developmental Neurobiology, Institute of Psychiatry, Psychology, and Neuroscience, King’s College London, London SE1 1UL, United Kingdom;; ^b^Medical Research Council Centre for Neurodevelopmental Disorders, King’s College London, London SE1 1UL, United Kingdom;; ^c^Department of Physiology, School of Medicine, University of California, San Francisco, CA 94143;; ^d^Department of Neurology, School of Medicine, University of California, San Francisco, CA 94143

**Keywords:** interneurons, striatum, cortex, cell death, cortico-striatal projections

## Abstract

The assembly of functional neuronal circuits requires appropriate numbers of distinct classes of neurons, but the mechanisms through which their relative proportions are established remain poorly defined. Investigating the mouse striatum, we found that the two most prominent subtypes of striatal interneurons, parvalbumin-expressing (PV^+^) GABAergic and cholinergic (ChAT^+^) interneurons, undergo extensive programmed cell death between the first and second postnatal weeks. Remarkably, the survival of PV^+^ and ChAT^+^ interneurons is regulated by distinct mechanisms mediated by their specific afferent connectivity. While long-range cortical inputs control PV^+^ interneuron survival, ChAT^+^ interneuron survival is regulated by local input from the medium spiny neurons. Our results identify input-specific circuit mechanisms that operate during the period of programmed cell death to establish the final number of interneurons in nascent striatal networks.

## Significance

Brain function requires appropriate numbers of different neuronal subtypes, but how these numbers are established remains poorly understood. Here, we identified a key role for the cerebral cortex in remotely controlling interneuron survival and thus establishing appropriate numbers of the two main types of interneurons in another brain region, the striatum. While cortical pyramidal cells directly control the survival of parvalbumin-expressing GABAergic neurons, the survival of cholinergic interneurons is indirectly controlled through the activity of the striatal medium spiny neurons. Our results demonstrate that the input-specific modulation of neuronal activity is universally required to control the survival of interneurons across very different brain circuits. This is important for the assembly of balanced neural networks.

There are hundreds of different types of projection neurons and interneurons in the mammalian nervous system, but the various cellular components of each brain structure arise during development in very precise proportions. This is largely achieved by an evolutionarily conserved strategy based on the initial overproduction of cells and the subsequent elimination of a fraction of them through the process of programmed cell death (1, 2).

In the neocortex, the relative proportion of excitatory pyramidal neurons and inhibitory GABAergic interneurons is similar across cortical areas and even species (3, 4). In mice, pyramidal cells and interneurons undergo extensive programmed cell death during early postnatal development to adjust their final ratio (5). Cortical interneurons appear to be intrinsically programmed to undergo cell death unless they sustain a certain level of activity during early postnatal development (6–9). This mechanism guarantees that only interneurons that receive sufficient input from pyramidal cells are retained (9, 10).

The striatum is another brain structure that contains both projection neurons and interneurons. However, it is unique in its almost complete lack of glutamatergic neurons. The principal neurons of the striatum, called medium spiny neurons (MSNs), are GABAergic and constitute 95% of all striatal neurons (11). Single-cell transcriptomic analyses have identified several populations of striatal interneurons in the mouse (12), including two major types for which there is abundant functional, morphological, and electrophysiological information: 1) parvalbumin-expressing (PV^+^) GABAergic interneurons and 2) choline acetyltransferase–expressing (ChAT^+^) cholinergic interneurons (13). Like many cortical interneurons, striatal PV^+^ and ChAT^+^ interneurons derive from progenitor cells in the medial ganglionic eminence (MGE) and preoptic area (POA) of the embryonic telencephalon (14), but it is presently unknown whether these cells also undergo programmed cell death during early postnatal development. In addition, the absence of local excitatory neurons raises the question of how the appropriate ratio of projection neurons and interneurons is established in the striatum and whether the cellular mechanisms underlying this process are universal across the brain. Here we found that striatal interneurons undergo extensive programmed cell death, a process that is specifically regulated by their afferent connectivity during a critical window of early postnatal development. Our experiments reveal that activity-dependent, input-specific control of programmed cell death regulates interneuron numbers in the striatum.

## Results

### Striatal Interneurons Undergo Programmed Cell Death.

**Fig. 1.**
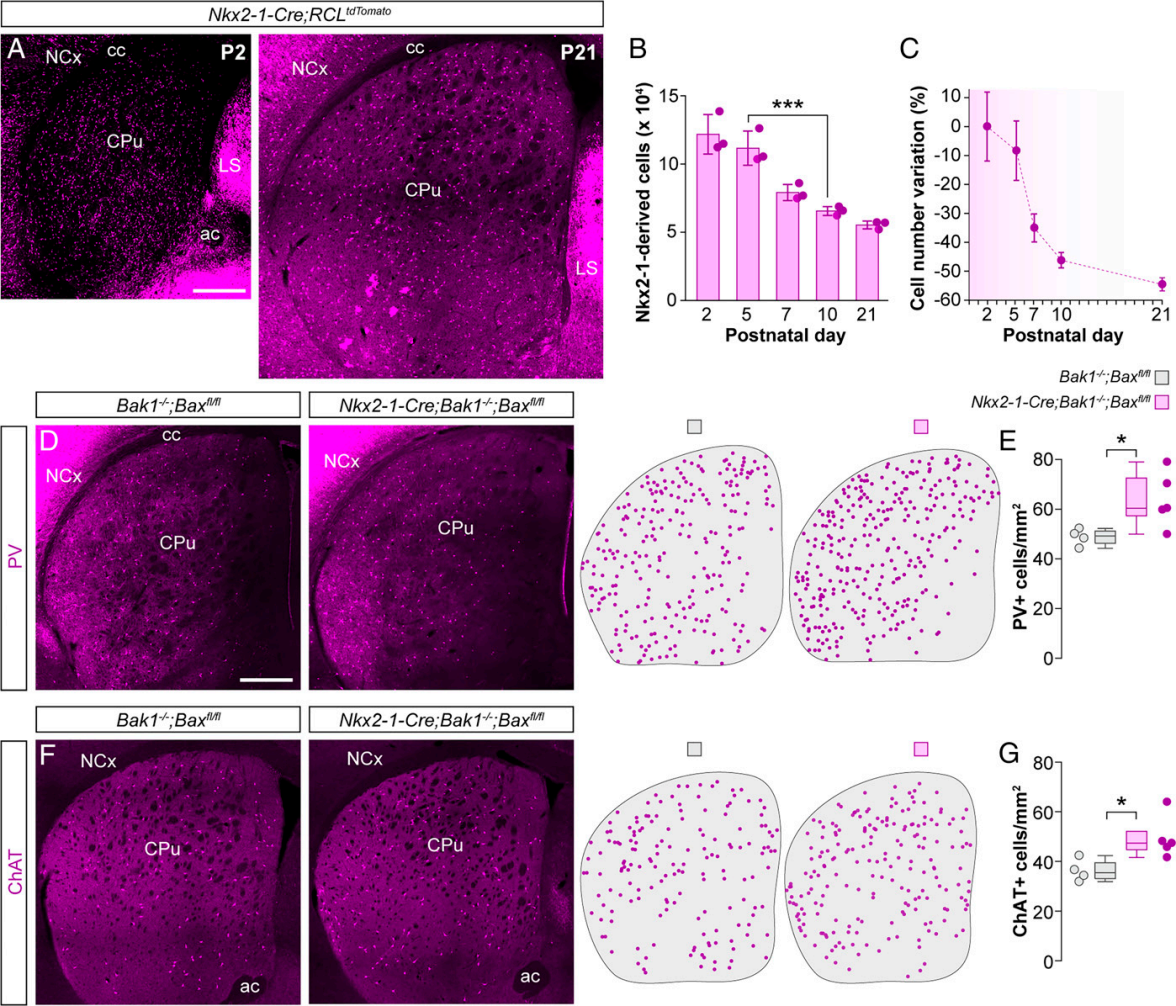
Striatal interneurons undergo programmed cell death during early postnatal development. (*A*) Coronal sections through the striatum of *Nkx2-1-Cre;RCL^tdTomato^* mice at P2 and P21. (*B*) Stereological estimates of the total number of tdTomato^+^ neurons in the striatum of *Nkx2-1-Cre;RCL^TdTomato^* mice at different postnatal stages (*n* = 3 mice per postnatal stage); one-way ANOVA, ***P < 0.001; P2 vs. P5: Tukey–Kramer HSD test, *P* > 0.05; P5 vs. P10: Tukey–Kramer HSD test, ***P < 0.001. (*C*) Percentage variation in the number of tdTomato^+^ cells at different postnatal stages relative to the average number of tdTomato^+^ cells at P2. (*D* and *F*) Coronal sections through the striatum of control *Bak1^−/−^;Bax^fl/fl^* and mutant *Nkx2-1-Cre;Bak1^−/−^;Bax^fl/fl^* mice immunostained for PV (*D*) and ChAT (*F*). The schematic dot maps indicate the locations of striatal interneurons in each case. (*E* and *G*) Quantification of PV^+^ (*E*) and ChAT^+^ (*G*) interneuron density in the striatum of control *Bak1^−/−^;Bax^fl/fl^* (*n* = 4) and mutant *Nkx2-1-Cre;Bak1^−/−^;Bax^fl/fl^* (*n* = 5) mice. PV^+^ interneurons: two-tailed unpaired Student’s *t* test, *P < 0.05. ChAT^+^ interneurons: Wilcoxon’s rank-sum test, *P < 0.05. Data in *B* are shown as bar plots and data in *E* and *G* as box plots. Adjacent data points indicate the stereological estimate in each mouse (*B*) or the average cell density in each animal (*E* and *G*). Error bars indicate SD. (Scale bars, 500 µm.).

To investigate whether striatal interneurons undergo programmed cell death during postnatal development, we generated *Nkx2-1-Cre;RCL^tdTomato^* mice in which MGE/POA-derived interneurons are irreversibly labeled from early embryonic development and quantified the number of tdTomato-expressing interneurons in the striatum using stereological methods. Between postnatal days P2 and P21 (Fig. 1*A*) we observed a dramatic decrease in the total number of striatal interneurons. We confirmed that striatal MGE/POA-derived interneurons undergo programmed cell death by staining for the apoptosis marker, cleaved caspase-3. Comparing P5 and P7, we observed a significant increase in the density of double-positive (Casp3^+^/tdTomato^+^) cells at P7 (*SI Appendix*, Fig. S1 *A* and *B*). By the end of the third postnatal week, the total number of MGE/POA-derived interneurons in the striatum reduced to nearly half the number found around birth, with the most significant variation occurring between P5 and P10 (Fig. 1 *B* and *C*).

We next sought to determine whether different populations of striatal MGE/POA-derived interneurons undergo programmed cell death at comparable rates. We hypothesized that like their cortical counterparts (6, 9), striatal interneurons might require the concerted action of the BCL2 family genes *Bax* and *Bak1* to undergo cell death. Consequently, removing *Bax* and *Bak1* in striatal interneurons would reveal how this process specifically affects distinct interneuron classes. To test this hypothesis, we generated *Nkx2-1-Cre;Bak1^−/−^;Bax^fl/fl^* mutant mice and quantified the density of PV^+^ and ChAT^+^ striatal interneurons. Suppressing cell death in MGE/POA-derived cells led to a ∼30% increase in the density of PV^+^ interneurons (Fig. 1 *D* and *E*) and a ∼36% increase in the density of ChAT^+^ interneurons (Fig. 1 *F* and *G*), indicating that both PV^+^ and ChAT^+^ interneuron numbers are strongly modulated during early postnatal development.

### Cortical Pyramidal Neurons Regulate Striatal Interneuron Survival.

**Fig. 2.**
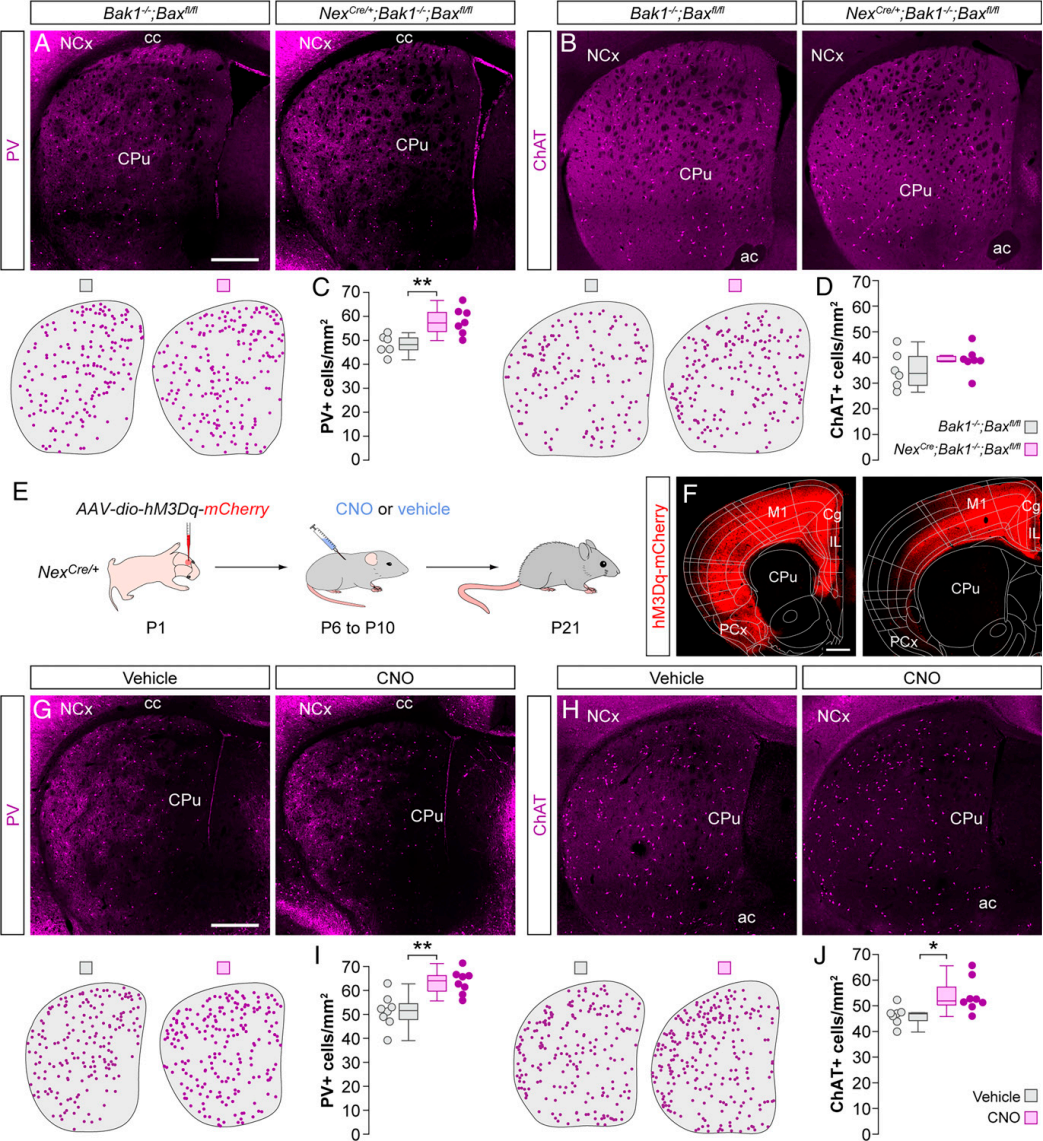
Cortical gain-of-function rescues striatal PV^+^ and ChAT^+^ interneurons. (*A* and *B*) Coronal sections through the striatum of control *Bak1^−/−^;Bax^fl/fl^* mice and mutant *Nex^Cre/+^;Bak1^−/−^;Bax^fl/fl^* mice immunostained for PV (*A*) and ChAT (*B*). The schematic dot maps indicate the locations of striatal interneurons in each case. (*C* and *D*) Quantification of PV^+^ (*C*) and ChAT^+^ (*D*) interneuron density in the striatum of control *Bak1^−/−^;Bax^fl/fl^* (*n* = 6) and mutant *Nex^Cre/+^;Bak1^−/−^;Bax^fl/fl^* (*n* = 7) mice. PV^+^ interneurons: two-tailed unpaired Student’s *t* test, **P < 0.01. ChAT^+^ interneurons: two-tailed unpaired Student’s *t* test, *P* = 0.27. (*E*) Schematic of experimental design. (*F*) Coronal sections through the brain at ∼1.5 mm (*Left*) and ∼0.5 mm (*Right*) anterior to Bregma reveal the extent of the viral infection. The images have been overlaid with the corresponding coronal maps. (*G* and *H*) Coronal sections through the striatum of vehicle- and CNO-treated *Nex^Cre/+^* mice immunostained for PV (*G*) and ChAT (*H*). The schematic dot maps indicate the locations of striatal interneurons in each case. (*I* and *J*) Quantification of PV^+^ (*I*) and ChAT^+^ (*J*) interneuron density in the striatum of vehicle (*n* = 8 for PV and 7 for ChAT)- and CNO-treated (*n* = 8) *Nex^Cre/+^* mice. PV^+^ interneurons: two-tailed unpaired Student’s *t* test, **P = 0.01. ChAT^+^ interneurons: two-tailed unpaired Student’s *t* test, *P < 0.05. Data in *C*, *D*, *I*, and *J* are shown as box plots and the adjacent data points indicate the average cell density in each animal. (Scale bars, 500 µm.).

The survival of cortical GABAergic interneurons is regulated by the number and activity of local excitatory neurons (9). The striatum lacks an equivalent population of local excitatory neurons, but its main source of excitatory input originates from the neocortex along with prominent input from the thalamus (15–17). To test whether long-range cortical inputs regulate striatal interneuron survival, we first asked whether preventing cortical excitatory neurons from undergoing programmed cell death—thereby artificially increasing their number—would, in turn, change the survival of striatal interneurons. To this end, we quantified the density of PV^+^ and ChAT^+^ interneurons in *Nex^Cre/+^;Bak1^−/−^;Bax^fl/fl^* mice, in which the programmed cell death of cortical pyramidal cells is specifically abolished (9). Artificially increasing the number of pyramidal neurons during development led to a ∼20% increase in the density of PV^+^ interneurons throughout the striatum (Fig. 2 *A* and *C*). In contrast, we observed no changes in the density of ChAT^+^ interneurons (Fig. 2 *B* and *D*).

The previous results revealed that the survival of striatal PV^+^ interneurons depends on the number of cortical pyramidal cells. PV^+^ interneurons preferentially receive synaptic input from corticostriatal projections (16), which might explain the differences observed among interneuron subtypes. Therefore, we investigated whether increasing the activity of cortical pyramidal cells during the period of striatal interneuron cell death would affect their survival. To this end, we transiently increased the activity of pyramidal cells using the chemogenetic actuator hM3Dq (18). We expressed an adeno-associated virus (AAV) encoding Cre-dependent hM3Dq-mCherry in the frontal cortex of *Nex^Cre^*^/+^ mice at P1 (Fig. 2 *E* and *F*). We then injected the pups with the ligand clozapine N-oxide (CNO) or vehicle twice daily between P6 and P10 and examined the distribution of striatal interneurons at P21. Enhancing pyramidal cell activity during the period of striatal interneuron cell death led to a robust ∼24% increase in the density of PV^+^ interneurons (Fig. 2 *G* and *I*) and a more modest increase of ∼17% in the density of ChAT^+^ interneurons (Fig. 2 *H* and *J*). To control for nonspecific effects of CNO that might arise through the activation of endogenous receptors (19), we injected a control AAV-encoding Cre-dependent mCherry in the frontal cortex of *Nex^Cre^*^/+^ mice at P1 (*SI Appendix*, Fig. S2*A*) and treated the injected pups with CNO or vehicle twice daily between P6 and P10. This manipulation did not significantly alter the density of either PV^+^ or ChAT^+^ interneurons in the striatum (*SI Appendix*, Fig. S2*B*).

To rule out the possibility that the observed increase in PV^+^ interneuron density is due to changes in PV levels and not in actual interneuron numbers, we quantified the density of striatal Nkx2-1^+^ cells that do not express ChAT. At P21, striatal Nkx2-1^+^/ChAT^−^ cells comprise all PV^+^ interneurons and a very small percentage of somatostatin neurons (14). We found an increase in Nkx2-1^+^/ChAT^−^ interneuron density in CNO-treated mice compared to controls (*SI Appendix*, Fig. S2 *D* and *E*), indicating that the increased density of striatal PV^+^ interneurons is likely due to increased survival and not to activity-dependent changes in PV levels.

### Paradoxical Effects of Cortical Dysfunction on Striatal Interneuron Survival.

**Fig. 3.**
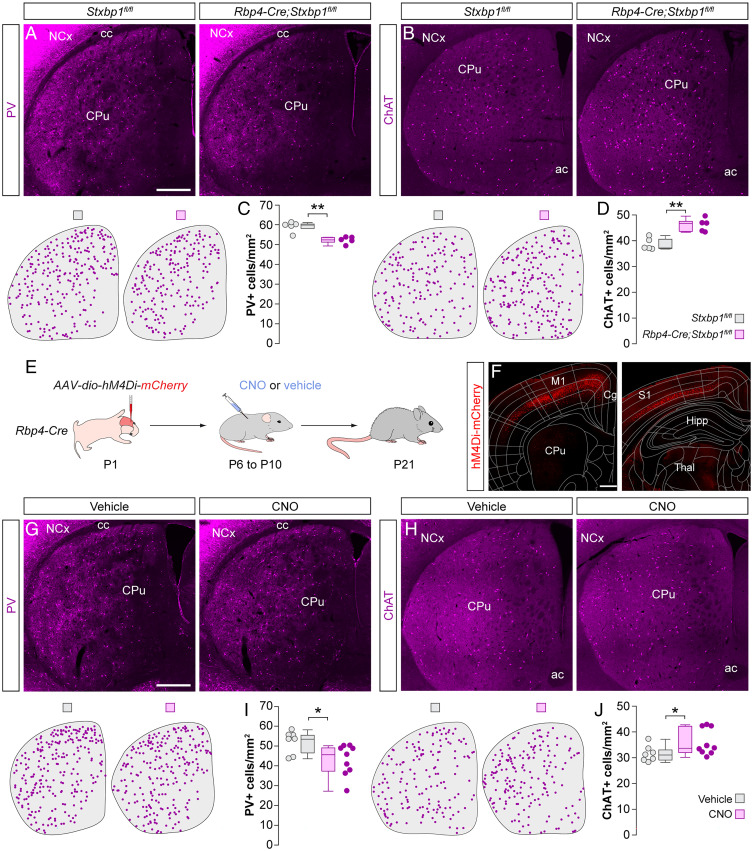
Cortical dysfunction differentially impacts the survival of striatal PV^+^ and ChAT^+^ interneurons. (*A* and *B*) Coronal sections through the striatum of control *Stxbp1^fl/fl^* mice and mutant *Rbp4-Cre;Stxbp1^fl/fl^* mice immunostained for PV (*A*) and ChAT (*B*). The schematic dot maps indicate the locations of striatal interneurons in each case. (*C* and *D*) Quantification of PV^+^ (*C*) and ChAT^+^ (*D*) interneuron density in the striatum of control *Stxbp1^fl/fl^* (*n* = 5) and mutant *Rbp4-Cre;Stxbp1^fl/fl^* (*n* = 5) mice. PV^+^ interneurons: Wilcoxon’s rank-sum test, **P < 0.01. ChAT^+^ interneurons: two-tailed unpaired Student’s *t* test, **P < 0.01. (*E*) Schematic of experimental design. (*F*) Coronal sections through the brain at ∼1 mm anterior (*Left*) and ∼2 mm posterior (*Right*) to Bregma reveal the extent of the viral infection. The images have been overlaid with the corresponding coronal maps. (*G* and *H*) Coronal sections through the striatum of vehicle- and CNO-treated *Rbp4-Cre* mice immunostained for PV (*G*) and ChAT (*H*). The schematic dot maps indicate the locations of striatal interneurons in each case. (*I* and *J*) Quantification of PV^+^ (*I*) and ChAT^+^ (*J*) interneuron density in the striatum of vehicle (*n* = 7)- and CNO-treated (*n* = 9) *Rbp4-Cre* mice. PV^+^ interneurons: two-tailed unpaired Student’s *t* test, *P < 0.05. ChAT^+^ interneurons: Wilcoxon’s rank-sum test, *P = 0.05. Data in *C*, *D*, *I*, and *J* are shown as box plots and the adjacent data points indicate the average cell density in each animal. (Scale bars, 500 µm.).

We next investigated whether the modulation of striatal interneuron numbers by cortical pyramidal neurons is bidirectional. Based on our previous results, we hypothesized that decreasing the number or activity of pyramidal neurons during the period of programmed cell death would negatively impact PV^+^ and ChAT^+^ interneuron survival. To test this hypothesis, we first generated *Rbp4-Cre;Stxbp1^fl/fl^* mice, in which syntaxin-binding protein 1 (also known as Munc18-1) is removed from Rbp4-expressing (Rbp4^+^) layer 5 pyramidal neurons in the neocortex. Deleting *Stxbp1* blocks neurotransmission, which is followed by rapid apoptosis of the cell (20). By P6, we found that mutant mice contained significantly fewer Ctip2^+^ layer 5 pyramidal cells in the primary somatosensory cortex (S1) and primary motor cortex (M1) than control mice (*SI Appendix*, Fig. S3 *A* and *B*). We then assessed whether the loss of corticostriatal projection neurons impacted the survival of striatal interneurons. Consistent with our hypothesis, we found that mutant mice contained ∼12% fewer striatal PV^+^ interneurons than controls at P21 (Fig. 3 *A* and *C*). Unexpectedly, we also observed an ∼20% increase in the density of striatal ChAT^+^ interneurons in mutants compared to control mice (Fig. 3 *B* and *D*).

Next, we tested whether decreasing the activity of pyramidal neurons has similar effects on striatal interneuron survival as reducing their number. We expressed Cre-dependent AAVs encoding the chemogenetic actuator hM4Di-mCherry (18) throughout the neocortex of *Rbp4-Cre* mice at P1 (Fig. 3 *E* and *F*). We then injected the pups with CNO or vehicle thrice daily between P6 and P10 and examined the distribution of striatal interneurons at P21. We found a significant decrease of ∼18% in the density of PV^+^ interneurons in CNO-treated mice compared to controls (Fig. 3 *G* and *I*). Interestingly, we again observed a significant increase of ∼14% in the density of striatal ChAT^+^ interneurons (Fig. 3 *H* and *J*). Altogether, these experiments demonstrated that the survival of striatal PV^+^ interneurons can be bidirectionally modulated by the number and activity of cortical pyramidal neurons. These experiments also revealed paradoxical effects on ChAT^+^ interneurons since both bidirectional manipulations led to an increase in the survival of this population.

### PV^+^ Interneuron Survival Requires Cortical Glutamatergic Input.

**Fig. 4.**
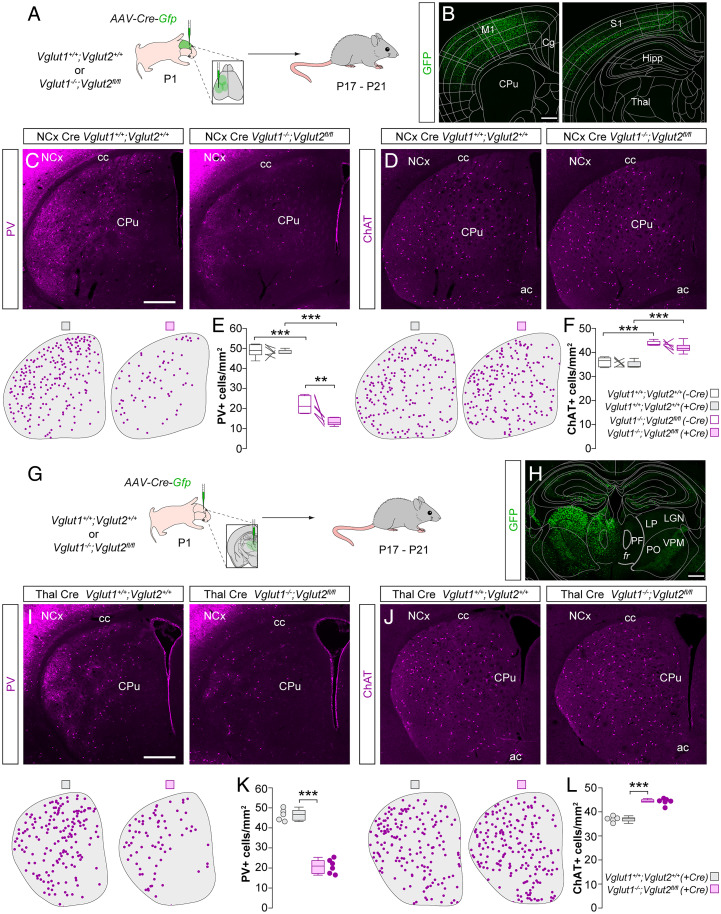
Long-range glutamatergic inputs regulate striatal interneuron survival. (*A*) Schematic of experimental design. (*B*) Coronal sections through the brain at ∼1 mm anterior (*Left*) and ∼2 mm posterior (*Right*) to Bregma reveal the extent of the viral infection. The images have been overlaid with the corresponding coronal maps. (*C* and *D*) Coronal sections through the striatum of Cre-injected control *Vglut1^+/+^;Vglut2^+/+^* and double-mutant *Vglut1^−/−^;Vglut2 ^fl/fl^* mice immunostained for PV (*C*) and ChAT (*D*). The schematic dot maps indicate the locations of striatal interneurons in each case. (*E* and *F*) Quantification of PV^+^ (*E*) and ChAT^+^ (*F*) interneuron density in the striatum of Cre-injected (+Cre) and uninjected (−Cre) hemispheres of control *Vglut1^+/+^;Vglut2^+/+^* (*n* = 6) and double-mutant *Vglut1^−/−^;Vglut2 ^fl/fl^* (*n* = 6) mice. PV^+^ interneurons: two-way ANOVA, ***P < 0.001; −Cre control vs. −Cre double mutant, Tukey–Kramer HSD test, ***P < 0.001; +Cre control vs. +Cre double mutant, Tukey–Kramer HSD test, ***P < 0.001; −Cre double mutant vs. +Cre double mutant, Tukey–Kramer HSD test, **P < 0.01. ChAT^+^ interneurons: two-way ANOVA, ***P < 0.001; −Cre control vs. −Cre double mutant, Tukey–Kramer HSD test, ***P < 0.001; +Cre control vs. +Cre double mutant, Tukey–Kramer HSD test, ***P < 0.001; −Cre double mutant vs. +Cre double mutant, Tukey–Kramer HSD test, *P* = 0.31. (*G*) Schematic of experimental design. (*H*) Coronal section through the brain at ∼2 mm posterior to Bregma reveals the extent of the viral infection. The image has been overlaid with the corresponding coronal map. (*I* and *J*) Coronal sections through the striatum of Cre-injected control *Vglut1^+/+^;Vglut2^+/+^* and double-mutant *Vglut1^−/−^;Vglut2 ^fl/fl^* mice immunostained for PV (*I*) and ChAT (*J*). The schematic dot maps indicate the locations of striatal interneurons in each case. (*K* and *L*) Quantification of PV^+^ (*K*) and ChAT^+^ (*L*) interneuron density in the striatum of Cre-injected (+Cre) hemispheres of control *Vglut1^+/+^;Vglut2^+/+^* (*n* = 5) and double-mutant *Vglut1^−/−^;Vglut2 ^fl/fl^* (*n* = 6) mice. PV^+^ interneurons: two-tailed unpaired Student’s *t* test, ***P < 0.001. ChAT^+^ interneurons: two-tailed unpaired Student’s *t* test, ***P < 0.001. Data in *E*, *F*, *K*, and *L* are shown as box plots and the adjacent data points indicate the average cell density in each animal. (Scale bars, 500 µm.) Abbreviations: FR, fasciculus retroflexus; PF, parafascicular nucleus; LP, lateral posterior nucleus; LGN, lateral geniculate nucleus; PO, posterior medial nucleus; VPM, ventral posteromedial nucleus.

In the neocortex, the activity of individual MGE interneurons correlates with their survival (9) and lowering their activity in a cell-autonomous manner reduces their ability to survive (10). Since the activity of any given neuron is largely derived from glutamatergic inputs, we tested whether striatal interneuron survival depends on glutamatergic neurotransmission from the cortex. To this end, we crossed double heterozygous *Vglut1* mutant and floxed *Vglut2* mice to obtain control (*Vglut1*^+/+^*; Vglut2^+/+^*) and double-mutant (*Vglut1*^−/−^*;Vglut2^fl/fl^*) animals. We deleted both vesicular glutamate transporters simultaneously because the loss of one of them can be functionally compensated by the other (21), and although Vglut1 is the main transporter expressed by cortical neurons, Vglut2 is transiently expressed in the cortex during the first week of postnatal development (22). Since previous work has demonstrated that disrupting glutamatergic transmission in the thalamus leads to aberrant cortical laminar development (21), we first checked whether deleting the glutamate transporters in the neocortex alters its cytoarchitecture. We observed no differences in the density and relative laminar distribution of NeuN^+^ cells in S1 and M1 (*SI Appendix*, Fig. S4 *A*–*C*). Next, we investigated the effects of deleting the vesicular glutamate transporters on the survival of striatal interneurons. We found that deletion of *Vglut1* alone (−Cre) leads to a ∼55% decrease in the density of striatal PV^+^ interneurons (Fig. 4*E*) by P17–P21. To additionally remove cortical *Vglut2* (+Cre), we made multiple injections of an AAV encoding Cre-GFP across the neocortex of control and double-mutant animals at P1 (Fig. 4 *A* and *B*). This led to a stronger decrease of ∼70% in the density of PV^+^ interneurons in the striatum (Fig. 4 *C* and *E*) as well as in the neocortex (*SI Appendix*, Fig. S4 *D* and *E*).

In contrast, the loss of cortical glutamatergic transmission led to a significant increase in the survival of striatal ChAT^+^ interneurons (Fig. 4 *D* and *F*). This increase did not differ between *Vglut1* deletion alone (−Cre) and the additional deletion of cortical *Vglut2* (+Cre), with *Vglut1* deletion leading to an ∼21% increase and *Vglut1/Vglut2* double deletion leading to an ∼20% increase. These results reveal that while glutamate released from corticostriatal neurons constitutes a crucial signal for the survival of striatal PV^+^ interneurons, it does not seem to act as a survival signal for ChAT^+^ interneurons. Indeed, the survival of ChAT^+^ interneurons increases in the absence of cortical glutamate.

### Thalamic Inputs Do Not Regulate PV^+^ or ChAT^+^ Interneuron Survival.

Having demonstrated that glutamatergic inputs from the cortex are essential for the survival of striatal PV^+^ but not ChAT^+^ interneurons, we next investigated the role of thalamic inputs in regulating striatal interneuron survival. In particular, ChAT^+^ interneurons are known to receive substantial inputs from the parafascicular nucleus of the thalamus (16, 23–25). Since thalamic neurons express *Vglut2* (26), to abolish glutamatergic transmission from the thalamus we injected AAVs encoding Cre-GFP into the midline thalamus of control (*Vglut1*^+/+^*; Vglut2^+/+^*) and double-mutant (*Vglut1*^−/−^*;Vglut2^fl/fl^*) mice at P1 (Fig. 4 *G* and *H*) and examined striatal interneuron densities at P17–P21 (Fig. 4 *I* and *J*). We observed an ∼55% decrease in the density of striatal PV^+^ interneurons (Fig. 4*K*). Interestingly, this percentage decrease is similar to the decrease observed when *Vglut1* is deleted alone (Fig. 4*E*), which indicates that the additional deletion of thalamic neurotransmission does not affect the survival of PV^+^ interneurons. Similarly, we observed an ∼20% increase in the density of striatal ChAT^+^ interneurons (Fig. 4*L*), which is similar to the percentage increase (∼21%) obtained when *Vglut1* alone is deleted (Fig. 4*F*). These results suggested that thalamic inputs to the striatum play a minimal role in regulating the survival of striatal PV^+^ and ChAT^+^ interneurons.

### ChAT^+^ Interneuron Survival Depends on MSN Activity.

**Fig. 5.**
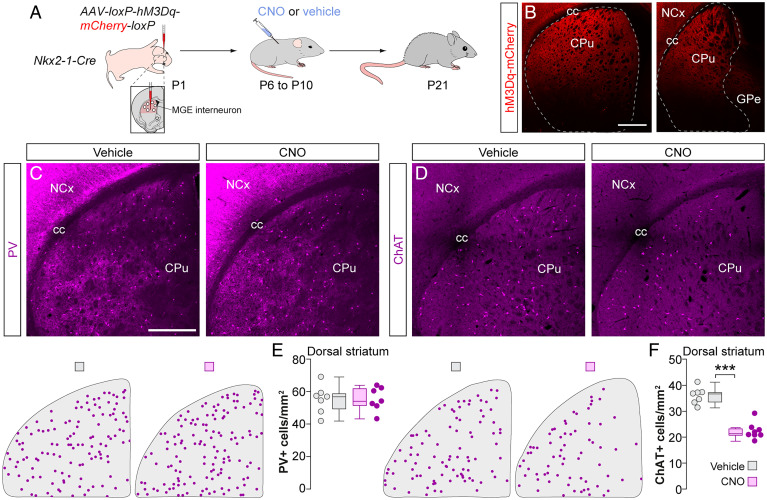
Local MSN inputs control striatal ChAT^+^ interneuron survival. (*A*) Schematic of experimental design. (*B*) Coronal sections through the striatum reveal the extent of the viral infection. Dashed lines indicate the boundary of the striatum. (*C* and *D*) Coronal sections through the dorsal striatum of vehicle- and CNO-treated *Nkx2-1-Cre* mice immunostained for PV (*C*) and ChAT (*D*). The schematic dot maps indicate the locations of striatal interneurons in each case. (*E* and *F*) Quantification of PV^+^ (*E*) and ChAT^+^ (*F*) interneuron density in the dorsal striatum of vehicle (*n* = 7)- and CNO-treated (*n* = 7 for PV and 8 for ChAT) *Nkx2-1-Cre* mice. PV^+^ interneurons: two-tailed unpaired Student’s *t* test, *P* = 0.97. ChAT^+^ interneurons: two-tailed unpaired Student’s *t* test, ***P < 0.001. Data in *E* and *F* are shown as box plots and the adjacent data points indicate the average cell density in each animal. (Scale bars, 500 µm.).

Striatal ChAT^+^ interneurons receive relatively weak direct inputs from pyramidal cells (16, 23), which suggests that the paradoxical effects on the survival of ChAT^+^ interneurons caused by manipulating the cortex might be indirect. We hypothesized that the survival of ChAT^+^ interneurons might be controlled by the activity of the MSNs since ChAT^+^ interneurons receive particularly strong inputs from these cells (27). We tested this hypothesis by injecting AAVs expressing hM3Dq-mCherry into the dorsal striatum of *Nkx2-1-Cre* mice at P1 (Fig. 5 *A* and *B*). To prevent the expression of hM3Dq in PV^+^ and ChAT^+^ interneurons, we flanked the expression construct with loxP sequences to excise it out in Cre-expressing populations (*SI Appendix*, Fig. S5 *A*–*C*). We then injected pups with CNO or vehicle thrice daily between P6 and P10 and examined the distribution of interneurons in the dorsal striatum at P21. Activating MSNs during the period of striatal interneuron cell death did not impact the survival of PV^+^ interneurons (Fig. 5 *C* and *E*). In contrast, we observed a prominent ∼38% decrease in the density of ChAT^+^ interneurons (Fig. 5 *D* and *F*), suggesting that MSN activity during this period negatively impacts the survival of ChAT^+^ interneurons.

## Discussion

Our study demonstrates that the neocortex can remotely influence the establishment of neural circuits in another region of the brain, the striatum. We found that the two most prominent types of striatal interneurons, PV^+^ GABAergic neurons and ChAT^+^ cholinergic neurons, undergo substantial programmed cell death during a short period of early postnatal development in the mouse. Their survival is under the control of specific afferent inputs during the cell death period. The final number of PV^+^ interneurons is established by long-range cortical inputs, while local inputs from medium spiny neurons regulate the final density of ChAT^+^ interneurons. Our results reveal circuit-based rules for establishing interneuron ratios and point to activity-dependent, input-specific mechanisms as the main determinant of the final number of interneurons in nascent striatal networks (*SI Appendix*, Fig. S6).

### Long-Range Control of Striatal PV^+^ Interneuron Survival.

Striatal PV^+^ interneurons mediate inhibition onto neighboring MSNs (28–30) and they are primarily driven by long-range excitatory inputs from the cortex (16, 31, 32). We found that modifying the number or activity of cortical pyramidal neurons bidirectionally regulates the survival of these interneurons. Given the key role these interneurons play in controlling MSN activity in response to cortical input (28–30, 33), it seems logical that pyramidal cells are directly involved in establishing their final numbers.

The majority of cortical inputs to the striatal PV^+^ interneurons originate from sensorimotor and frontal-association areas (15, 32, 34). These include the orbital, insular, limbic, and cingulate cortices (15). Consistent with this observation, chemogenetic activation of the frontal cortex leads to a robust ∼24% increase in the density of PV^+^ interneurons. These experiments broadly targeted the frontal cortex, including motor as well as frontal-association regions. Not all of these regions may participate equally in regulating the survival of PV^+^ interneurons. Further experiments, using more fine-scale cortical manipulations, are required to elucidate the specific contribution of different cortical regions to the survival of PV^+^ interneurons.

Genetic ablation and chemogenetic inactivation of layer 5 corticostriatal neurons led to a significant but more modest reduction in the survival of striatal PV^+^ cells. In particular, we observed only an ∼12% decrease in PV^+^ interneuron density in *Rbp4-Cre;Stxbp1^fl/fl^* mice, compared to littermate controls. We note that this ablation strategy does not account for all layer 5 excitatory neurons. At P6, we obtained a 15% and 42% reduction in the density of Ctip2^+^ cells in S1 and M1 cortex, respectively, indicating that the majority of layer 5 excitatory neurons persist following this manipulation. It is conceivable that the inputs from the remaining layer 5 neurons are sufficient to rescue the majority of striatal PV^+^ interneurons from programmed cell death. Alternatively, other ectopic regions, or cortical layers such as layer 2/3, which has been shown to project to the striatum (35), might compensate for the reduced corticostriatal input. The neocortex controls the survival of striatal PV^+^ interneurons through glutamatergic neurotransmission. Preventing exocytotic glutamate release from pyramidal cells in large parts of the dorsal neocortex, including frontal, motor, and somatosensory cortices, led to the elimination of nearly 70% of striatal PV^+^ interneurons.

PV^+^ interneurons of the striatum also receive excitatory inputs from the parafascicular nucleus of the thalamus (16, 31, 36). Surprisingly, disrupting glutamate release from the parafascicular nucleus did not impact the survival of these interneurons. It is conceivable that thalamic inputs to these interneurons might not be fully functional during the period of programmed cell death, which might explain the minimal role of the thalamus in controlling their survival. Finally, striatal PV^+^ interneurons are not influenced by the local activity of their main target, the neighboring MSNs, presumably because they receive relatively few inputs from these cells (27, 37).

### Local Control of Striatal ChAT^+^ Interneuron Survival.

ChAT^+^ interneurons of the striatum receive glutamatergic inputs from the frontal cortex and the parafascicular nucleus of the thalamus (15, 16, 23–25, 38). Eliminating glutamate release from large parts of the dorsal neocortex, including frontal, motor, and somatosensory cortices, led to an ∼20% increase in the density of these interneurons. Furthermore, disrupting glutamatergic transmission from the thalamus did not affect their survival. These results indicate that striatal ChAT^+^ interneurons do not depend on either cortical or thalamic glutamate as a survival signal. On the contrary, these interneurons show better survival when cortical excitatory neurons are either ablated, their activity dampened, or glutamate release from the cortex is abolished altogether.

The final number of striatal ChAT^+^ interneurons is primarily regulated by the activity of the local MSNs. Increasing MSN activity negatively impacts ChAT^+^ interneuron survival. This result is consistent with the observation that ChAT^+^ interneurons receive prominent inputs from these cells (27, 38, 39). The neocortex can influence the survival of striatal ChAT^+^ cells, although this effect is likely mediated by the indirect modulation of MSN activity. Several lines of evidence support this conclusion. First, pyramidal cells provide only weak and sparse direct excitatory inputs to ChAT^+^ interneurons (16, 23). Second, in the absence of excitatory synaptic drive, MSNs remain quiescent due to a very hyperpolarized resting membrane potential (40). Consequently, in experiments where corticostriatal inputs are reduced, it is expected that MSN activity would decrease, which would in turn increase the survival of ChAT^+^ cells. Third, since the number of striatal PV^+^ interneurons scale up when the number or activity of pyramidal neurons are experimentally raised, increasing corticostriatal drive may paradoxically decrease the activity of MSNs and indirectly increase the survival of ChAT^+^ interneurons.

It is tempting to speculate on the source of the signals promoting the survival of ChAT^+^ interneurons. One possibility is that glutamate release from an unknown source might control their survival. In this context, we note that a recent study discovered an excitatory projection from the pedunculopontine nucleus to striatal ChAT^+^ interneurons (15). Alternatively, other striatal neurotransmitters such as dopamine and serotonin might play a role in regulating their survival. Finally, it has been shown that ChAT^+^ interneurons are tonically active (41, 42). This spontaneous activity is generated by endogenous ionic conductances (43, 44), independently of excitatory synapses (43, 45). It is therefore conceivable that the endogenous firing of striatal ChAT^+^ interneurons might promote the survival of these cells during the period of programmed cell death.

### Outlook.

Striatal interneuron dysfunction is associated with neurodevelopmental disorders that affect movement, cognition, and behavior. Alterations in striatal interneuron numbers have been described in patients with Tourette syndrome and schizophrenia (46–48). Equally, experimental manipulation of the activity and number of these cells leads to motor stereotypies and other functional deficits in mice (49–51). In the striatum, the regulation of PV^+^ interneuron numbers is important for establishing balanced ratios of excitation and inhibition. Furthermore, this balance appears to be crucial for the survival of ChAT^+^ interneurons. Elucidating whether similar mechanisms operate in humans may shed new light on the neurobiology of neurodevelopmental disorders with alterations in the number of interneurons.

## Methods

The mouse lines *Nex^Cre/+^* [*Neurod6^tm1(cre)Kan^*], *Nkx2-1-Cre* [*Tg(Nkx2-1-cre)2Sand*], *RCL^tdTomato^* [*Gt(ROSA)26Sor^tm9(CAG-tdTomato)Hze^*], *Bak^−/−^,Bax^fl/fl^* (*Bak1^tm1Thsn^* and *Bax^tm2Sjk^*), *Rbp4-Cre* [*Tg(Rbp4-cre)KL100Gsat*], *Stxbp1^fl/fl^* (*Stxbp1^tm1MVer^*), and *Vglut1^−/−^;Vglut2^fl/fl^* (*Slc17a7^tm1Edw^* and *Slc17a6^tm1.1Thna^*) were used in this study. Animals were housed in groups of up to five littermates and maintained under standard, temperature-controlled, laboratory conditions. Mice were kept on a 12:12 light/dark cycle and received water and food ad libitum. All animal procedures were approved by the ethical committee (King’s College, London) and conducted following European regulations and Home Office personal and project licenses under the UK Animals (Scientific Procedures) 1986 Act. A full description of methods can be found in *SI Appendix*.

## Supplementary Material

Supplementary File

## Data Availability

Raw imaging data and MATLAB analysis files have been deposited on the King’s Open Research Data System and can be accessed at doi: 10.18742/19222449. Any reagents that this study generated will be shared by the last author upon reasonable request. All other study data are included in the article and/or supporting information.
