# Supplementary material for: Input-specific control of interneuron numbers in nascent striatal networks
Source: Proc Natl Acad Sci U S A. 2022 May 9;119(20):e2118430119. doi: 10.1073/pnas.2118430119 (PMC9171775; doi:10.1073/pnas.2118430119)
Supplement: Supplementary File [file pnas.2118430119.sapp.pdf]

Supplementary Information for

Input-specific control of interneuron numbers in nascent striatal networks

Varun Sreenivasan[a,b], Eleni Serafeimidou-Pouliou[a,b], David Exposito-Alonso[a,b], Kinga Bercsenyi[a,b], Clémence Bernard[a,b], Sung-Eun Bae[a,b], Fazal Oozeer[a,b], Alicia Hanusz-Godoy[a,b], Robert Edwards[c] and Oscar Marín[a,b,1]

[a]Centre for Developmental Neurobiology, Institute of Psychiatry, Psychology and Neuroscience, King's College London, London SE1 1UL, United Kingdom; [b]MRC Centre for Neurodevelopmental Disorders, King's College London, London SE1 1UL, United Kingdom; [c]Department of Physiology and Department of Neurology, School of Medicine, University of California San Francisco, United States of America

This file includes:

Supplementary Figures 1 to 6
Materials and Methods
Supplementary references
Supplementary Table 1

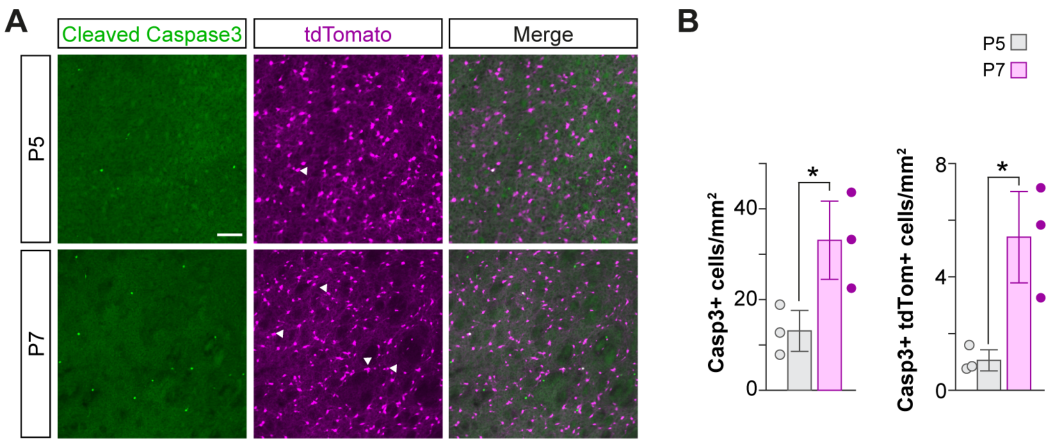

Supplementary Figure 1

Supplementary Fig. 1. Increase in striatal cleaved Caspase-3 positive cells between P5 and P7. **(A)** Coronal sections through the striatum of *Nkx2-1-Cre;RCL*^{tdTomato} mice, immunostained for cleaved Caspase-3, at P5 and P7. White arrowheads indicate double positive cells. **(B)** Quantification of Casp3+ and Casp3+/tdTomato+ cell density in the striatum at P5 (n = 3) and P7 (n = 3). Total Caspase-3 cell density: two-tailed unpaired Student's t-test, *$p < 0.05$. Double positive cell density: two-tailed unpaired Student's t-test, *$p < 0.05$. Data are shown as bar plots and adjacent data points indicate the average cell density in each animal. Error bars indicate standard deviation. Scale bar, 100 μm.

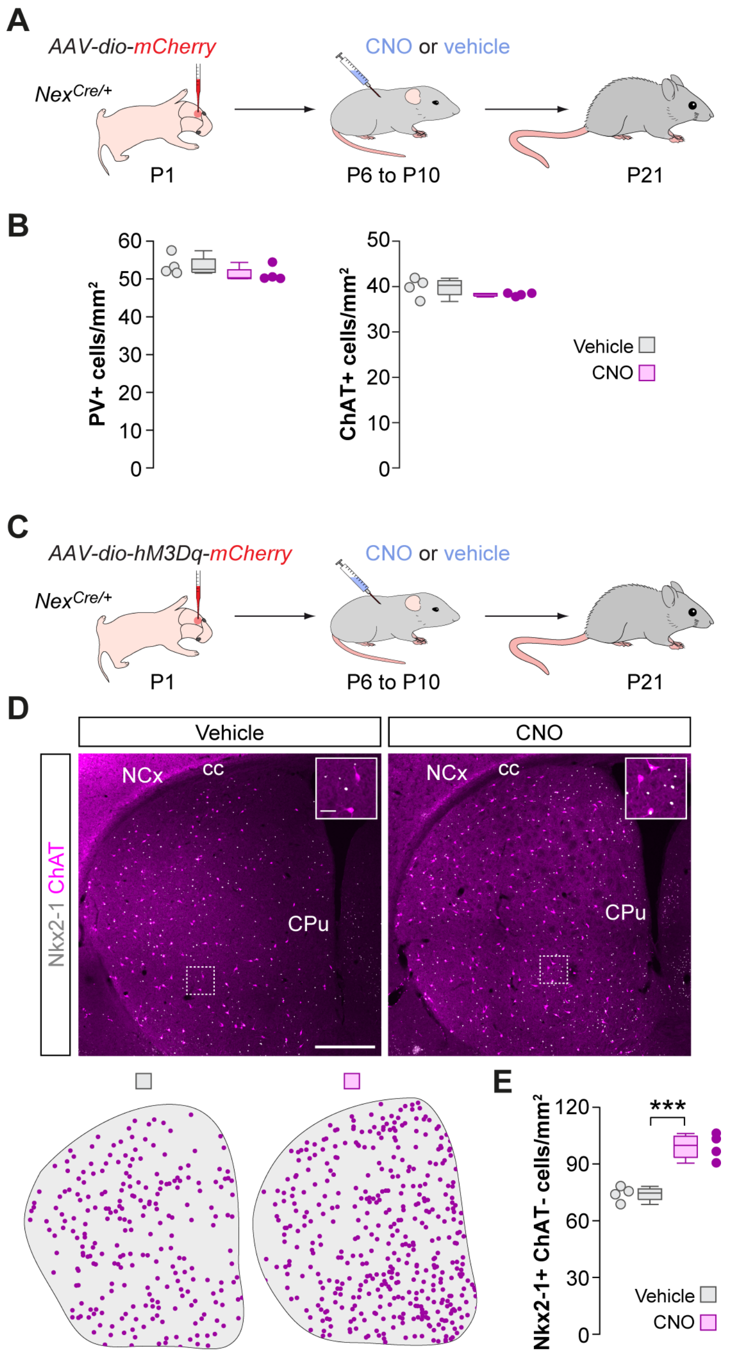

Supplementary Figure 2

Supplementary Fig. 2. The increase in the density of PV+ interneurons is due to rescue from cell death and not to activity-related changes in PV+ expression. **(A)** Schematic of experimental design. **(B)** Quantification of PV+ and ChAT+ interneuron density in the striatum of vehicle (n = 4) and CNO-treated (n = 4) $Nex^{Cre/+}$ mice, injected with AAV expressing *hSyn-DIO-mCherry* in frontal cortex. PV+ interneurons: two-tailed unpaired Student's t-test, $p = 0.23$. ChAT+ interneurons: two-tailed unpaired Student's t-test, $p = 0.20$ **(C)** Schematic of experimental design. **(D)** Coronal sections through the striatum of vehicle and CNO treated $Nex^{Cre/+}$ mice, injected with AAV expressing *hSyn-DIO-hM3D(Gq)-mCherry* in the frontal cortex, immunostained for Nkx2-1 and ChAT. The schematic dot maps indicate the locations of Nkx2-1+/ChAT- interneurons in each case. **(E)** Quantification of Nkx2-1+/ChAT- interneuron (putative PV+ cell) density in the striatum of vehicle (n = 4) and CNO treated (n = 4) $Nex^{Cre/+}$ mice. Two-tailed unpaired Student's t-test, $^{***}p < 0.001$. Data are shown as boxplots and adjacent data points indicate the average cell density in each animal. Scale bars, 500 μm and 50 μm (inset).

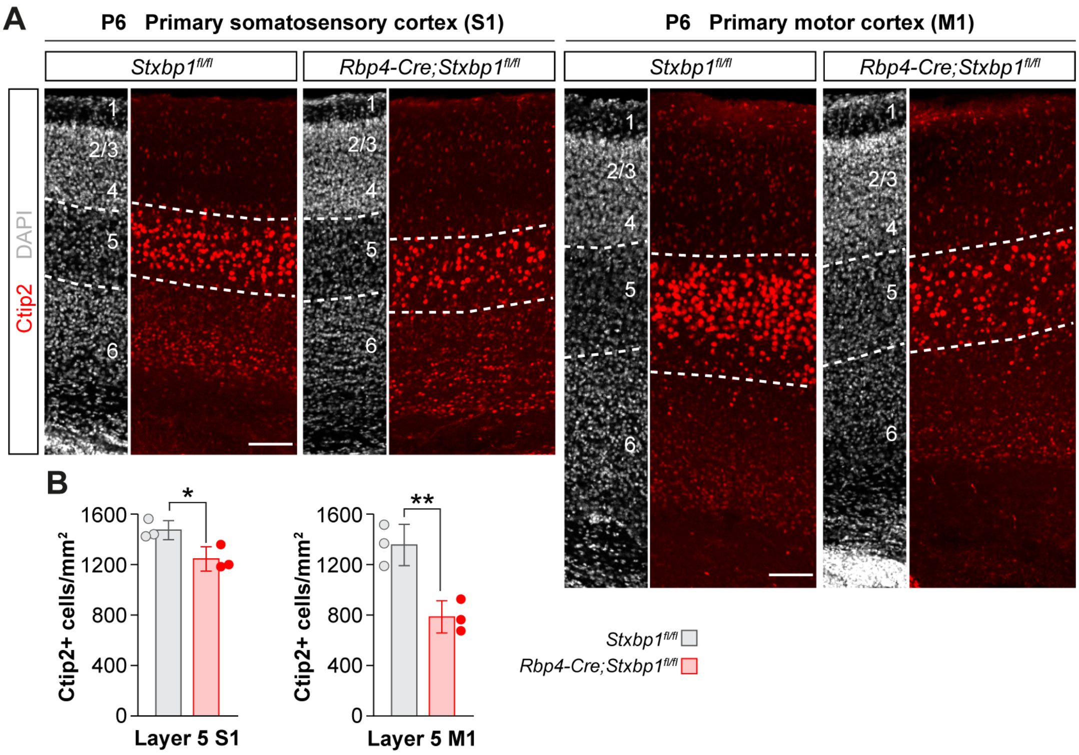

Supplementary Figure 3

Supplementary Fig. 3. Deleting *Stxbp1* causes the death of layer 5 neurons by postnatal day 6. (A) Coronal sections through primary somatosensory cortex (S1) and primary motor cortex (M1) of control *Stxbp1*$^{fl/fl}$ and *Rbp4-Cre;Stxbp1*$^{fl/fl}$ mice immunostained for Ctip2 at P6. **(B)** Quantification of layer 5 Ctip2+ cell density in S1 and M1 of control *Stxbp1*$^{fl/fl}$ (n = 3) and mutant *Rbp4-Cre;Stxbp1*$^{fl/fl}$ (n = 3) mice. S1: two-tailed unpaired Student's t-test, $*p < 0.05$. M1: two-tailed unpaired Student's t-test, $**p < 0.01$. Data are shown as bar plots and adjacent data points indicate the average cell density in each animal. Error bars indicate standard deviation. Scale bar, 100 μm.

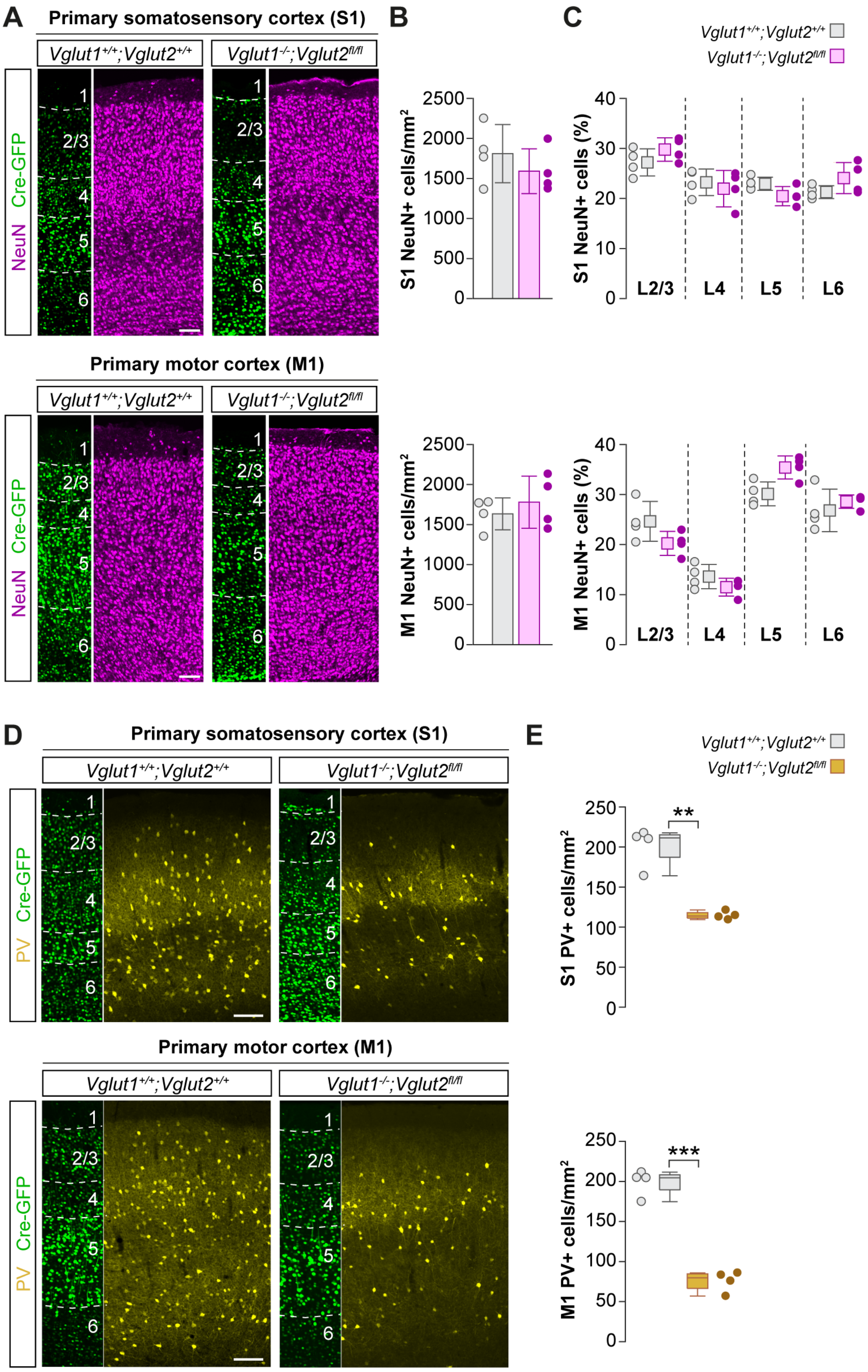

Supplementary Figure 4

Supplementary Fig. 4. Abolishing glutamatergic transmission in the cortex does not change cortical lamination but impacts the survival of cortical PV+ interneurons. (A) Coronal sections through primary somatosensory cortex (S1) and primary motor cortex (M1) of AAV *hSyn-Cre-GFP* injected control *Vglut1*$^{+/+}$*;Vglut2*$^{+/+}$ and double-mutant *Vglut1*$^{-/-}$*;Vglut2*$^{fl/fl}$ mice, immunostained for the pan-neuronal marker NeuN. **(B)** Quantification of total NeuN+ cell density in S1 and M1 of control *Vglut1*$^{+/+}$*;Vglut2*$^{+/+}$ (n = 4) and double-mutant *Vglut1*$^{-/-}$*;Vglut2*$^{fl/fl}$ (n = 4) mice. S1: two-tailed unpaired Student's t-test, $p = 0.37$; M1: two-tailed unpaired Student's t-test, $p = 0.47$. **(C)** Percentage of NeuN+ cells in different layers of control *Vglut1*$^{+/+}$*;Vglut2*$^{+/+}$ (n = 4) and double-mutant *Vglut1*$^{-/-}$*;Vglut2*$^{fl/fl}$ (n = 4) mice. S1: two-way ANOVA with Tukey-Kramer HSD test: L2/3, $p = 0.82$; L4, $p = 0.99$; L5, $p = 0.85$; L6, $p = 0.75$. M1: two-way ANOVA with Tukey-Kramer HSD test: L2/3, $p = 0.36$; L4, $p = 0.95$; L5, $p = 0.17$; L6, $p = 0.98$. **(D)** Coronal sections through primary somatosensory cortex and primary motor cortex of AAV *hSyn-Cre-GFP* injected control *Vglut1*$^{+/+}$*;Vglut2*$^{+/+}$ and double-mutant *Vglut1*$^{-/-}$*;Vglut2*$^{fl/fl}$ mice, immunostained for PV. **(E)** Quantification of PV+ interneuron density in S1 and M1 of control *Vglut1*$^{+/+}$*;Vglut2*$^{+/+}$ (n = 4) and double-mutant *Vglut1*$^{-/-}$*;Vglut2*$^{fl/fl}$ (n = 4) mice. S1: two-tailed unpaired Student's t-test, $^{**}p < 0.01$; M1: two-tailed unpaired Student's t-test, $^{***}p < 0.001$. Data are shown as bar plots (panel B) and boxplots (panel E). Adjacent data points indicate the average cell density in each animal. Error bars in panels B and C indicate standard deviation Scale bar, 100 μm.

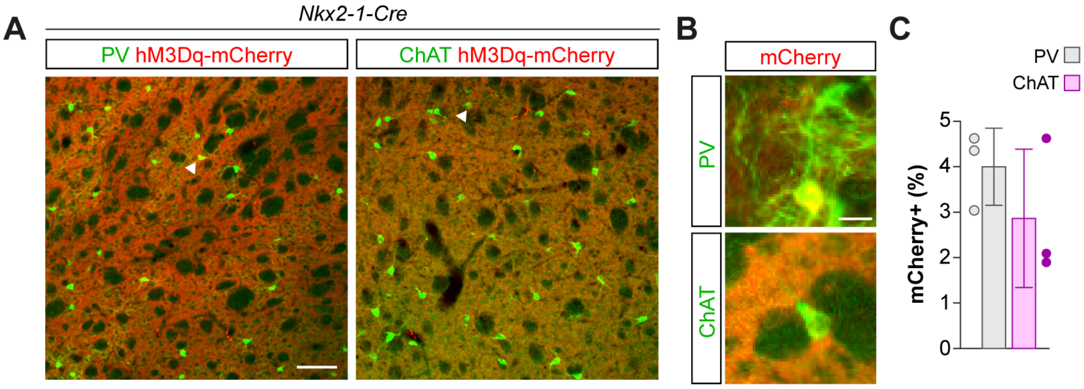

Supplementary Figure 5

Supplementary Fig. 5. Quantification of double positive cells in *Nkx2-1-Cre* mice injected with AAV *hSyn-loxP-hM3d(Gq)-mCherry-loxP*. (A) Coronal sections through the striatum of *Nkx2-1-Cre* mice, injected with *hSyn-loxP-hM3d(Gq)-mCherry-loxP* in the striatum, immunostained for PV and ChAT. White arrowheads indicate double positive cells. **(B)** High-magnification images of PV and ChAT cells expressing mCherry. **(C)** Quantification of the percentage of PV+ and ChAT+ interneurons within the injection site expressing mCherry**.** Data are shown as bar plots and adjacent data points indicate the average cell density in each animal. Error bars indicate standard deviation. Scale bars, 100 μm (panel A) and 20 μm (panel B).

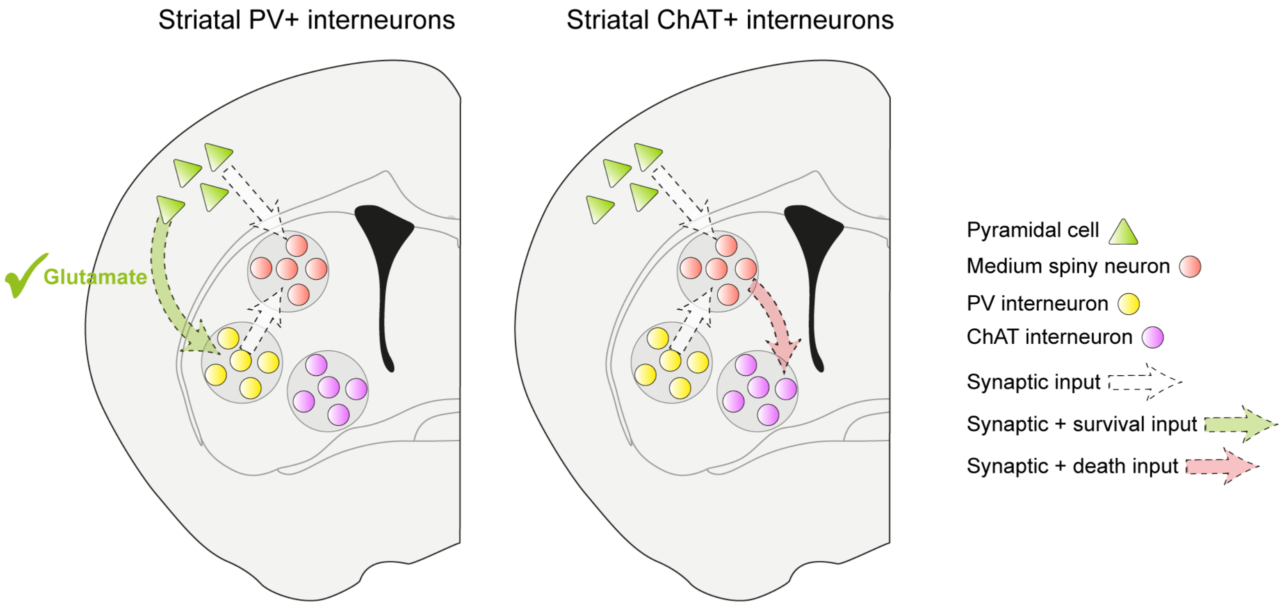

Supplementary Figure 6

Supplementary Fig. 6. Circuit mechanisms regulating programmed cell death of striatal interneurons. Striatal PV+ interneurons receive glutamatergic input from cortical pyramidal neurons that rescue them from programmed cell death and help to establish appropriate levels of inhibition in the striatum (left). The activity of striatal medium spiny neurons (MSNs) is tightly regulated by a combination of excitatory glutamatergic inputs from the cortex and inhibitory GABAergic inputs from PV+ interneurons. An imbalance leading to hyperactivity in MSNs kills ChAT+ interneurons (right).

Materials and Methods

Mice. The mouse lines $Nex^{Cre/+}$ ($Neurod6^{tm1(cre)Kan}$) (1), *Nkx2-1-Cre* (*Tg(Nkx2-1-cre)2Sand*) (2), RCL^{tdT} ($Gt(ROSA)26Sor^{tm9(CAG\text{-}tdTomato)Hze}$) (3), $Bak^{-/-}$,$Bax^{fl/fl}$ ($Bak1^{tm1Thsn}$ and Bax^{tm2Sjk}) (4), *Rbp4-Cre* (*Tg(Rbp4-cre)KL100Gsat*) (5), $Stxbp1^{fl/fl}$ ($Stxbp1^{tm1MVer}$) (6), $Vglut1^{-/-}$;$Vglut2^{fl/fl}$ ($Slc17a7^{tm1Edw}$ and $Slc17a6^{tm1.1Thna}$) (7, 8) were used in this study. Animals were housed in groups of up to five littermates and maintained under standard, temperature-controlled, laboratory conditions. Mice were kept on a 12:12 light/dark cycle and received water and food ad libitum. All animal procedures were approved by the ethical committee (King's College London) and conducted following European regulations, and Home Office personal and project licenses under the UK Animals (Scientific Procedures) 1986 Act.

Generation of DNA constructs. The Addgene plasmid *pAAV hSyn-hM3D(Gq)-mCherry* (Addgene 50474) (9) was used as a starting point to generate *pAAV hSyn-lox-hM3D(Gq)-mCherry-lox*. Addgene_50474 was first digested with Sal1 and EcoR1 to generate a 4545 bp backbone for further cloning. The following primers were used to PCR amplify a fragment of 2597 bp containing *hM3D(Gq)-mCherry* flanked by *loxP* sites in the same directional orientation: Fw primer: 5'CTAGAGTCGACATAACTTCGTATAGCATACATTATAC GAAGTTATGCCACCATGACCTTGCAC-3'. Rv primer: 5'TATCGAATTCATAACTT CGTATAATGTATGCTATACGAAGTTATTTACTTGTACAGCTCGTCC-3'.

The fragments were purified using QIAquick gel extraction kit (QIAGEN, Cat# 28704) The PCR amplified fragment was digested with Sal1 and EcoR1 and ligated with the backbone to give the final construct.

AAV production. AAVs were produced using polyethylenimine (PEI) transfection of HEK293FT cells as described before (10). In brief, DNA and PEI were mixed in the ratio of 1:4 in uncomplemented DMEM and left at room temp for 25 mins to form the DNA-PEI complex. The transfection solution was added to each plate and incubated for 72 h at 37 ºC in 5% CO2. The transfected cells were then scraped off the plates and pelleted. The cell pellet was lysed in buffer containing 50mN Tris-Cl, 150mM NaCl and 2mM MgCl2 and 0.5% sodium deoxycholate and incubated with 100U/ml Benzonase (Sigma-Aldrich, Cat# E1014 25KU) for 1 h to dissociate particles from membranes. Any remaining contaminants and empty or incomplete viral particles were removed with discontinuous iodixanol (OptiPrepTM) (Sigma-Aldrich, Cat# D1556) gradient ultracentrifugation using four layers of different iodixanol concentrations of 15, 25, 40 and 58% (11). The viral suspension was loaded on the iodixanol gradient in Quick-seal polyallomer tubes (Beckman-Coulter, Cat# 342414) and spun in a VTi-50 rotor at 50,000 rpm for 75 mins at 12ºC in an Optima L-100 XP Beckman Coulter ultracentrifuge. The recovered virus fraction was purified by first passing through a 100-kDa molecular weight cut off (MWCO) centrifugal filter (Sartorius Vivaspin, Cat# VS2041) and then through an Amicon Ultra 2ml Centrifugal filters (Merck Millipore, Cat# UFC210024). Storage buffer (350 mM NaCl and 5% Sorbitol in PBS) was added to the purified virus and stored in 5 μl aliquots at -80 ºC. AAV titer was determined by quantitative polymerase chain reaction (qPCR) using primers for the WPRE sequence that is common to the construct. The following primers were used: WPRE Forward primer: 5'GGCACTGACAATTCCGTGGT-3'. WPRE Reverse primer: 5'-CGCTGGATTGAGGGCCGAAG-3'. The extracted viral DNA and a serial dilution of the transfer plasmid DNA containing the WPRE sequence were transferred to a 96 well plate and measured using a LightCycler® 96 instrument (Roche Life Science). AAVs produced had a titer of 2.10 x 10^{13} viral genomes/ml.

Stereology. The total number of striatal MGE interneurons was estimated using the optical dissector method (12)

$$N = \frac{\sum Q^{-}.t}{h\,.\,asf.ssf}$$

where ΣQ^- is the total number of cells counted, t the mean section thickness, h the height of the optical dissector (18 μm) adjusting for the guard zones (2 μm) above and below the dissector, *asf* stands for the area sampling fraction and *ssf* stands for the section sampling fraction (sampling frequency). An ApoTome (Zeiss) microscope equipped with a motorized stage and color camera was connected to a computer with the Stereo Investigator software (MBF Biosciences). The boundaries of the striatum were first defined with a 2.5x objective (Zeiss) and the entire area was split into 4 equal quadrants.

Intracranial injections. P1 mice were anesthetized with 3% isofluorane and mounted on a stereotaxic frame. Isoflurane concentration was maintained between 1 and 2% throughout the procedure. Injections of *hSyn-DIO-hM3D(Gq)-mCherry* and *hSyn-DIO-mCherry* were targeted to the frontal cortex, at ~ 0.5 mm anterior and 1 mm lateral to bregma, of the left hemisphere. 600 nl of virus was injected at a depth of 400 μm in the left hemisphere at a speed of 3 nl sec^{-1}. Injections of *hSyn-DIO-hM4D(Gi)-mCherry* were targeted to 6 sites in the left hemisphere to infect layer 5 neurons in the entire cortex. At each site, 200 nl of virus was injected at a depth of 600 μm and a speed of 3 nl sec^{-1}. Injections of *hSyn-Cre-GFP* were targeted to 6 sites in the left hemisphere to infect neurons in the entire cortex. At each site, 200 nl of virus was injected at a depth of 400 μm and a speed of 3 nl sec^{-1}. Thalamic injections of *hSyn-GFP-Cre* were targeted to a location lying 3.2 to 3.3 mm posterior to the blood vessel lying immediately behind the olfactory bulb and ~0.5 mm lateral to the midline. 700 nl of virus was injected at a depth of ~1.9 to 2.1 mm and at a speed of 3 nl sec^{-1}. Injections of *hSyn-lox-hM3D(Gq)-mCherry-lox* were targeted to a single site in the left striatum. Along the anteroposterior axis, the site was located halfway between bregma and the blood vessel lying immediately behind the olfactory bulb and at ~1.3 to 1.5 mm lateral to the midline. 600 nl of virus was injected at a depth of ~1.4 to 1.6 mm and at a speed of 3 nl sec^{-1}, to infect neurons in the dorsal striatum.

CNO injections. Injected animals were randomly assigned to the "Vehicle" or "CNO" groups. Clozapine-N-oxide (Tocris Bioscience, Cat# 4936) was dissolved in 0.9% saline and 0.5% DMSO (Sigma-Aldrich, Cat# D8418) to give a final concentration of 0.5 mg ml^{-1}. The vehicle consisted of 0.5% DMSO in saline without the active ligand. In experiments where *hSyn-DIO-hM3D(Gq)-mCherry* or *hSyn-DIO-mCherry* was expressed in the frontal cortex, CNO or vehicle was administered subcutaneously twice a day from P6 until P10 at a dose of 1 mg kg^{-1}. This period was chosen since P6 and P10 correspond to the start and end of striatal interneuron death, respectively. In experiments where *hSyn-lox-hM3D(Gq)-mCherry-lox* was expressed in the striatum, CNO or vehicle was administered subcutaneously thrice a day from P6 until P10 at a dose of 5 mg kg^{-1}. In experiments where *hSyn-DIO-hM4D(Gi)-mCherry* was expressed in layer 5 across the entire cortex, CNO or vehicle was administered subcutaneously thrice a day from P6 until P10 at a dose of 5 mg kg^{-1}.

Histology and immunohistochemistry. Mice were deeply anesthetized with sodium pentobarbital (Euthatal, Merial Animal Health Ltd) by intraperitoneal injection, and were transcardially perfused with 0.9% NaCl solution (Sigma-Aldrich, Cat# S76530) followed by 4% paraformaldehyde (PFA) (Sigma-Aldrich, Cat# 441244) dissolved in phosphate-buffered saline (PBS). Brains were extracted and post-fixed for 2h at 4°C. The brains were immersed

overnight in 30% sucrose (Sigma-Aldrich, Cat# S0389) and coronal sections were cut frozen on a sliding microtome (Leica SM2010 R) to a thickness of 40 μm. Every 8th section was used for antibody staining. The sampling was therefore every 280 μm. Free-floating brain slices were permeabilized with 0.25% Triton X-100 (Sigma-Aldrich, Cat# T8787) in PBS for 30 mins, and blocked for 1h in a blocking buffer containing 0.25% Triton X-100, 10% normal donkey serum (Sigma-Aldrich, Cat# S30), and 5% bovine serum albumin (BSA) (Sigma-Aldrich, Cat# A8806). Primary antibodies were dissolved in fresh blocking buffer and the sections were incubated overnight at 4°C in the primary antibody solution. The following day, the tissue was repeatedly rinsed. Secondary antibodies were dissolved in fresh blocking buffer and the sections were incubated in the secondary antibody solution for 2h at room temperature. The sections were counterstained with 5 μM 4',6-diamidine-2'-phenylindole dihydrochloride (DAPI) (Sigma-Aldrich, Cat# D9542) in PBS, rinsed repeatedly and mounted on a glass slide with Mowiol and DABCO. The following primary antibodies were used: Rabbit anti-parvalbumin (1:1000, Swant, Cat# PV-27), goat anti-ChAT (1:500, Millipore, Cat# AB144P), rabbit anti-TTF1 (1:500, Abcam, Cat# ab76013), rat anti-Ctip2 (1:1000, Abcam, Cat# ab18465), rabbit anti-cleaved Caspase-3 (1:200, Cell Signaling Technology, Cat# 9661) and mouse anti-NeuN (1:300, Millipore, Cat# MAB377). The following secondary antibodies were used: Donkey-anti-rabbit-Alexa 555 (1:400, Molecular Probes, Cat# 31572), donkey anti-rabbit-Alexa 488 (1:400, Thermo Fisher Scientific, Cat# 21206), donkey anti-rabbit-Alexa 647 (1:400, Thermo Fisher Scientific, Cat# 31573), donkey-anti-goat Alexa 488 (1:400, Molecular Probes, Cat# 11055), donkey-anti-goat Alexa 555 (1:400, Invitrogen, Cat# 21432), donkey-anti-goat Alexa 647 (1:400, Thermo Fisher Scientific, Cat# 21447), donkey-anti-rat Cy3 (1:400, Jackson ImmunoResearch Europe Ltd., Cat# 712-165-150) and donkey-anti-mouse Alexa 647 (1:400, Thermo Fisher Scientific, Cat# 31571).

Image acquisition and image analysis. Tile scan images of the striatum were acquired at 1,024x1,024-pixel resolution and 8-bit depth on an inverted Leica TCS-SP8 confocal microscope. 5 to 8 sections covering the striatum along the rostrocaudal axis were imaged. Samples from the same experimental litter were imaged and analyzed in parallel using similar laser powers, photomultiplier gain and detection filter settings. Images were analyzed using custom routines written in MATLAB. Briefly, images were first adjusted for brightness and contrast. A median filter was applied to the image to remove salt-and-pepper noise followed by Sobel filtering to determine the edges of the cells. This was followed by the following morphological operations: one round of dilation (*imdilate* function), one round of filling (*imfill* function) and two rounds of erosion (*imerosion* function). The cell positions were calculated as the centroids (*regionprops* function). The striatum was delineated and the number of centroids inside it was counted and divided by the total area of the region to give the cell density in cells per mm^2. This analysis was repeated for all sections where the striatum was present and individual values for the sections were averaged to give a single value per animal.

Statistical analysis. All statistical analyses were performed using MATLAB (Supplementary Table 1). Group data are presented as boxplots. On each box, the central mark indicates the median and the top and bottom edges of the box indicate the 25th and 75th percentiles respectively. The whiskers extend to the data points not considered as outliers. Outliers were considered as points lying at 1.5 x IQR (inter-quartile range) above the 75th percentile or under the 25th percentile. The Anderson-Darling normality test was used to compare the empirical distribution of the data sets with a normal probability distribution. To compare normally distributed data, we used paired or unpaired two-tailed Student's t-test. To compare non-normally distributed data, we used Wilcoxon's rank-sum test for unpaired comparisons or Wilcoxon's signed-rank test for paired comparisons respectively. To analyze the

differences among multiple experimental groups, we used a one-way analysis of variance (ANOVA) followed by the Tukey-Kramer post-hoc test. Statistical significance was considered at p-values ≤ 0.05. The number of animals for each experiment is described in each figure legend.

Supplementary References

1. S. Goebbels *et al.*, Genetic targeting of principal neurons in neocortex and hippocampus of NEX-Cre mice. *Genesis* **44**, 611-621 (2006).
2. Q. Xu, M. Tam, S. A. Anderson, Fate mapping Nkx2.1-lineage cells in the mouse telencephalon. *J. Comp. Neurol.* **506**, 16-29 (2008).
3. L. Madisen *et al.*, A robust and high-throughput Cre reporting and characterization system for the whole mouse brain. *Nat. Neurosci.* **13**, 133-140 (2010).
4. O. Takeuchi *et al.*, Essential role of BAX,BAK in B cell homeostasis and prevention of autoimmune disease. *Proc. Natl. Acad. Sci. USA* **102**, 11272-11277 (2005).
5. C. R. Gerfen, R. Paletzki, N. Heintz, GENSAT BAC cre-recombinase driver lines to study the functional organization of cerebral cortical and basal ganglia circuits. *Neuron* **80**, 1368-1383 (2013).
6. J. H. Heeroma *et al.*, Trophic support delays but does not prevent cell-intrinsic degeneration of neurons deficient for munc18-1. *Eur. J. Neurosci.* **20**, 623-634 (2004).
7. R. T. Fremeau, Jr. *et al.*, Vesicular glutamate transporters 1 and 2 target to functionally distinct synaptic release sites. *Science* **304**, 1815-1819 (2004).
8. T. S. Hnasko *et al.*, Vesicular glutamate transport promotes dopamine storage and glutamate corelease in vivo. *Neuron* **65**, 643-656 (2010).
9. B. L. Roth, DREADDs for Neuroscientists. *Neuron* **89**, 683-694 (2016).
10. E. Favuzzi *et al.*, Distinct molecular programs regulate synapse specificity in cortical inhibitory circuits. *Science* **363**, 413-417 (2019).
11. S. Zolotukhin *et al.*, Recombinant adeno-associated virus purification using novel methods improves infectious titer and yield. *Gene Ther* **6**, 973-985 (1999).
12. M. J. West, H. J. Gundersen, Unbiased stereological estimation of the number of neurons in the human hippocampus. *J. Comp. Neurol.* **296**, 1-22 (1990).

Supplementary Table 1. Summary of data and statistical analyses for Figures 1-5 and Supplementary Figures 1-5.

FIGURE 1	Measurement	Values	N	Statistical test	p-value
Figure 1B	MGE interneurons in whole striatum (mean ± s.d.)	P2: 121774 ± 14518; P5: 111609 ± 12512; P7: 79111 ± 5921; P10: 65528 ± 3260; P21: 55326 ± 2921	[brains] *Nkx2-1-Cre; RCL^{tdTomato}*: P2, n = 3; P5, n = 3; P7, n = 3; P10, n = 3; P21, n = 3	One-way ANOVA with Tukey-Kramer HSD test	P2 vs. P5, $p > 0.05$; P5 vs. P10, $p < 0.001$ (***)
Figure 1C	% change in MGE interneurons (mean ± s.d.)	P2: 0 ± 12; P5: -8.3 ± 10.3; P7: -35.0 ± 4.9; P10: -46.2 ± 2.7; P21: -54.5 ± 2.4	[brains] *Nkx2-1-Cre; RCL^{tdTomato}*: P2, n = 3; P5, n = 3; P7, n = 3; P10, n = 3; P21, n = 3		
Figure 1E	PV cell density (cells/mm^2)	*Bak1$^{-/-}$; Bax$^{fl/fl}$* : mean = 48.7, median = 49.2, inter-quartile range = 4.9; *Nkx2-1-Cre; Bak1$^{-/-}$; Bax$^{fl/fl}$* : mean = 63.9, median = 60.4, inter-quartile range = 15.2. % change = +31.1%	[brains] *Bak1$^{-/-}$; Bax$^{fl/fl}$*, n = 4; *Nkx2-1-Cre; Bak1$^{-/-}$; Bax$^{fl/fl}$*, n = 5	Unpaired *t*-test	$p < 0.05$ (*)
Figure 1G	ChAT cell density (cells/mm^2)	*Bak1$^{-/-}$; Bax$^{fl/fl}$* : mean = 36.2, median = 35.4, inter-quartile range = 6.4; *Nkx2-1-Cre; Bak1$^{-/-}$; Bax$^{fl/fl}$* : mean = 49.3, median = 47.3, inter-quartile range = 7.4. % change = +36.0%	[brains] *Bak1$^{-/-}$; Bax$^{fl/fl}$*, n = 4; *Nkx2-1-Cre; Bak1$^{-/-}$; Bax$^{fl/fl}$*, n = 5	Wilcoxon's rank-sum test	$p < 0.05$ (*)
**FIGURE 2**	**Measurement**	**Values**	**N**	**Statistical test**	**p-value**
Figure 2C	PV cell density (cells/mm^2)	*Bak1$^{-/-}$; Bax$^{fl/fl}$* : mean = 48.0, median = 48.2, inter-quartile range = 5.0; *Nex$^{Cre/+}$; Bak1$^{-/-}$; Bax$^{fl/fl}$* : mean = 57.9, median = 57.2, inter-quartile range = 8.0. % change = +20.5%	[brains] *Bak1$^{-/-}$; Bax$^{fl/fl}$*, n = 6; *Nex$^{Cre/+}$; Bak1$^{-/-}$; Bax$^{fl/fl}$*, n = 7	Unpaired *t*-test	$p < 0.01$ (**)
Figure 2D	ChAT cell density (cells/mm^2)	*Bak1$^{-/-}$; Bax$^{fl/fl}$* : mean = 35.0, median = 33.8, inter-quartile range = 11.3; *Nex$^{Cre/+}$; Bak1$^{-/-}$; Bax$^{fl/fl}$* : mean = 39.0, median = 38.7, inter-quartile range = 2.0. % change = +11.3%	[brains] *Bak1$^{-/-}$; Bax$^{fl/fl}$*, n = 6; *Nex$^{Cre/+}$; Bak1$^{-/-}$; Bax$^{fl/fl}$*, n = 7	Unpaired *t*-test	$p = 0.27$
Figure 2I	PV cell density (cells/mm^2)	*hM3Dq (vehicle)* : mean = 51.0, median = 51.4, inter-quartile range = 6.6; *hM3Dq (CNO)* : mean = 63.2, median = 63.9, inter-quartile range = 6.5. % change = +23.8%	[brains] *hM3Dq* (*Vehicle*), n = 8; *hM3Dq* (*CNO*), n = 8	Unpaired *t*-test	$p = 0.01$ (**)
Figure 2J	ChAT cell density (cells/mm^2)	*hM3Dq (vehicle)* : mean = 46.1, median = 46.9, inter-quartile range = 3.0; *hM3Dq (CNO)* : mean = 53.8, median = 52.0, inter-quartile range = 7.0. % change = +16.7%	[brains] *hM3Dq* (*Vehicle*), n = 7; *hM3Dq* (*CNO*), n = 8	Unpaired *t*-test	$p < 0.05$ (*)
**FIGURE 3**	**Measurement**	**Values**	**N**	**Statistical test**	**p-value**
Figure 3C	PV cell density (cells/mm^2)	*Stxbp1$^{fl/fl}$* : mean = 58.9, median = 59.8, inter-quartile range = 2.3; *Rbp4-Cre; Stxbp1$^{fl/fl}$* : mean = 52.1, median = 52.3, inter-quartile range = 2.3. % change = -11.5%	[brains] *Stxbp1$^{fl/fl}$*, n = 5; *Rbp4-Cre; Stxbp1$^{fl/fl}$*, n = 5	Wilcoxon's rank-sum test	$p < 0.01$ (**)

Figure 3D	ChAT cell density (cells/mm^2)	*Stxbp1*$^{fl/fl}$: mean = 38.6, median = 37.2, inter-quartile range = 3.7; *Rbp4-Cre; Stxbp1*$^{fl/fl}$: mean = 46.1, median = 46.8, inter-quartile range = 4.0. % change = +19.4%	[brains] *Stxbp1*$^{fl/fl}$, n = 5; *Rbp4-Cre; Stxbp1*$^{fl/fl}$, n = 5	Unpaired *t*-test	$p < 0.01$ (**)
Figure 3I	PV cell density (cells/mm^2)	*hM4Di (vehicle)* : mean = 52.0, median = 53.5, inter-quartile range = 8.9; *hM4Di (CNO)* : mean = 42.8, median = 45.7, inter-quartile range = 11.7. % change = -17.7%	[brains] *hM4Di* (*Vehicle*), n = 7; *hM4Di* (*CNO*), n = 9	Unpaired *t*-test	$p < 0.05$ (*)
Figure 3J	ChAT cell density (cells/mm^2)	*hM4Di (vehicle)* : mean = 31.4, median = 31.0, inter-quartile range = 3.9; *hM4Di (CNO)* : mean = 35.8, median = 33.6, inter-quartile range = 10.2. % change = +14.0%	[brains] *hM4Di* (*Vehicle*), n = 7; *hM4Di* (*CNO*), n = 9	Wilcoxon's rank-sum test	$p = 0.05$ (*)
**FIGURE 4**	**Measurement**	**Values**	**N**	**Statistical test**	**p-value**
Figure 4E	PV cell density (cells/mm^2)	*Vglut1*$^{+/+}$*;Vglut2*$^{+/+}$ (-Cre) : mean = 48.8, median = 49.0, inter-quartile range = 5.0; *Vglut1*$^{-/-}$*;Vglut2*$^{fl/fl}$ (-Cre) : mean = 21.8, median = 21.1, inter-quartile range = 8.7. % change = -55.3%; *Vglut1*$^{+/+}$*;Vglut2*$^{+/+}$ (+Cre) : mean = 47.8, median = 47.6, inter-quartile range = 1.5; *Vglut1*$^{-/-}$*;Vglut2*$^{fl/fl}$ (+Cre): mean = 13.3, median = 12.8, inter-quartile range = 3.5. % change = -72.2%	[brains] *Vglut1*$^{+/+}$*;Vglut2*$^{+/+}$, n = 6; *Vglut1*$^{-/-}$*;Vglut2*$^{fl/fl}$, n = 6	Two-way ANOVA with Tukey-Kramer HSD test	$p < 0.001$ (***)
			[brains] *Vglut1*$^{+/+}$*;Vglut2*$^{+/+}$, n = 6; *Vglut1*$^{-/-}$*;Vglut2*$^{fl/fl}$, n = 6	Two-way ANOVA with Tukey-Kramer HSD test	$p < 0.001$ (***)
Figure 4F	ChAT cell density (cells/mm^2)	*Vglut1*$^{+/+}$*;Vglut2*$^{+/+}$ (-Cre): mean = 36.1, median = 36.6, inter-quartile range = 3.7; *Vglut1*$^{-/-}$*;Vglut2*$^{fl/fl}$ (-Cre): mean = 43.8, median = 43.3, inter-quartile range = 1.6. % change = +21.2%; *Vglut1*$^{+/+}$*;Vglut2*$^{+/+}$ (+Cre): mean = 35.0, median = 34.2, inter-quartile range = 2.2; *Vglut1*$^{-/-}$*;Vglut2*$^{fl/fl}$ (+Cre): mean = 42.0, median = 41.6, inter-quartile range = 2.1. % change = +20.2%	[brains] *Vglut1*$^{+/+}$*;Vglut2*$^{+/+}$, n = 6; *Vglut1*$^{-/-}$*;Vglut2*$^{fl/fl}$, n = 6	Two-way ANOVA with Tukey-Kramer HSD test	$p < 0.001$ (***)
			[brains] *Vglut1*$^{+/+}$*;Vglut2*$^{+/+}$, n = 6; *Vglut1*$^{-/-}$*;Vglut2*$^{fl/fl}$, n = 6	Two-way ANOVA with Tukey-Kramer HSD test	$p < 0.001$ (***)
Figure 4K	PV cell density (cells/mm^2)	*Vglut1*$^{+/+}$*;Vglut2*$^{+/+}$ (+Cre) : mean = 46.5, median = 46.7, inter-quartile range = 4.9; *Vglut1*$^{-/-}$*;Vglut2*$^{fl/fl}$ (+Cre): mean = 20.8, median = 21.0, inter-quartile range = 6.4. % change = -55.3%	[brains] *Vglut1*$^{+/+}$*;Vglut2*$^{+/+}$, n = 5; *Vglut1*$^{-/-}$*;Vglut2*$^{fl/fl}$, n = 6	Unpaired *t*-test	$p < 0.001$ (***)
Figure 4L	ChAT cell density (cells/mm^2)	*Vglut1*$^{+/+}$*;Vglut2*$^{+/+}$ (+Cre): mean = 36.9, median = 37.1, inter-quartile range = 1.3; *Vglut1*$^{-/-}$*;Vglut2*$^{fl/fl}$ (+Cre): mean = 44.2, median = 44.4, inter-quartile range = 1.0. % change = +19.8%	[brains] *Vglut1*$^{+/+}$*;Vglut2*$^{+/+}$, n = 5; *Vglut1*$^{-/-}$*;Vglut2*$^{fl/fl}$, n = 6	Unpaired *t*-test	$p < 0.001$ (***)

FIGURE 5	Measurement	Values	N	Statistical test	p-value
Figure 5E	PV cell density (cells/mm^2)	*lox_{OUT}-hM3Dq- lox_{OUT} (vehicle)* : mean = 55.1, median = 56.8, inter-quartile range = 9.3; *lox_{OUT}-hM3Dq- lox_{OUT} (CNO)* : mean = 55.3, median = 54.0, inter-quartile range = 10.3. % change = +0.3%	[brains] *lox_{OUT}-hM3Dq- lox_{OUT}* (*Vehicle*), n = 7; *lox_{OUT}-hM3Dq- lox_{OUT}* (*CNO*), n = 7	Unpaired *t*-test	p = 0.97
Figure 5F	ChAT cell density (cells/mm^2)	*lox_{OUT}-hM3Dq- lox_{OUT} (vehicle)* : mean = 35.9, median = 36.7, inter-quartile range = 3.6; *lox_{OUT}-hM3Dq- lox_{OUT} (CNO)*: mean = 22.2, median = 21.3, inter-quartile range = 2.5. % change = -38.0%	[brains] *lox_{OUT}-hM3Dq- lox_{OUT}* (*Vehicle*), n = 7; *lox_{OUT}-hM3Dq- lox_{OUT}* (*CNO*), n = 8	Unpaired *t*-test	p < 0.001 (***)

FIGURE S1	Measurement	Values	N	Statistical test	p-value
Figure S1B	Casp3+ cell density (cells/mm^2)	*P5*: mean = 13.1, median =12.6, inter-quartile range = 8.3; *P7*: mean = 33.0, median = 33.2, inter-quartile range = 15.9	[brains] *P5*, n = 3; *P7*, n = 3	Unpaired *t*-test	p < 0.05 (*)
	Casp3+tdTomato+ cell density (cells/mm^2)	*P5*: mean = 1.0, median = 0.8, inter-quartile range = 0.6; *P7*: mean = 5.4, median = 5.8, inter-quartile range = 2.9	[brains] *P5*, n = 3; *P7*, n = 3	Unpaired *t*-test	p < 0.05 (*)

FIGURE S2	Measurement	Values	N	Statistical test	p-value
Figure S2B	PV cell density (cells/mm^2)	*mCherry (vehicle)* : mean = 53.5, median = 52.5, inter-quartile range = 3.5; *mCherry (CNO)* : mean = 51.3, median = 50.3, inter-quartile range = 2.3. % change = -4.2%	[brains] *mCherry* (*Vehicle*), n = 4; *mCherry* (*CNO*), n = 4	Unpaired *t*-test	p = 0.23
	ChAT cell density (cells/mm^2)	*mCherry (vehicle)* : mean = 39.7, median = 40.2, inter-quartile range = 3.0; *mCherry (CNO)* : mean = 38.2, median = 38.3, inter-quartile range = 0.6. % change = -4.0%	[brains] *mCherry* (*Vehicle*), n = 4; *mCherry* (*CNO*), n = 4	Unpaired *t*-test	p = 0.20
Figure S2E	Nkx2.1$^+$ChAT$^-$ cell density (cells/mm^2)	*hM3Dq (vehicle)*: mean = 74.0, median = 74.6, inter-quartile range = 5.6; *hM3Dq (CNO)*: mean = 99.1, median = 99.9, inter-quartile range = 11.2. % change = +33.9%	[brains] *hM3Dq* (*Vehicle*), n = 4; *hM3Dq* (*CNO*), n = 4	Unpaired *t*-test	p < 0.001 (***)

FIGURE S3	Measurement		Values	N	Statistical test	p-value
Figure S3B	Layer 5 Ctip$^+$ cell density (cells/ mm^2)	S1 (mean ± s.d.)	*Stxbp1$^{fl/fl}$* : 1473.0 ± 75.8; *Rbp4-Cre; Stxbp1$^{fl/fl}$* : 1244.5 ± 96.9	[brains] *Stxbp1$^{fl/fl}$*, n = 3; *Rbp4-Cre; Stxbp1$^{fl/fl}$*, n = 3	Unpaired *t*-test	p < 0.05 (*)
		M1 (mean ± s.d.)	*Stxbp1$^{fl/fl}$* : 1355.3 ± 163.8; *Rbp4-Cre; Stxbp1$^{fl/fl}$* : 785.7 ± 127.5	[brains] *Stxbp1$^{fl/fl}$*, n = 3; *Rbp4-Cre; Stxbp1$^{fl/fl}$*, n = 3	Unpaired *t*-test	p < 0.01 (**)

FIGURE S4	Measurement		Values	N	Statistical test	p-value
Figure S4B	NeuN+ cell density (cells/mm^2)	S1 (mean ± s.d.)	*Vglut1$^{+/+}$;Vglut2$^{+/+}$* : 1809.4 ± 363.6 *Vglut1$^{-/-}$;Vglut2$^{fl/fl}$* : 1590.6 ± 279.4	[brains] *Vglut1$^{+/+}$;Vglut2$^{+/+}$*, n = 4; *Vglut1$^{-/-}$;Vglut2$^{fl/fl}$*, n = 4	Unpaired *t*-test	p = 0.37

		M1 (mean ± s.d.)	$Vglut1^{+/+};Vglut2^{+/+}$: 1633.2 ± 199.0<br>$Vglut1^{-/-};Vglut2^{fl/fl}$: 1778.9 ± 326.0	[brains] $Vglut1^{+/+};Vglut2^{+/+}$, n = 4; $Vglut1^{-/-};Vglut2^{fl/fl}$, n = 4	Unpaired *t*-test	*p* = 0.47
Figure S4C	NeuN+ layer percentage	S1 (mean ± s.d.)	$Vglut1^{+/+};Vglut2^{+/+}$: L2/3, 27.2 ± 2.7; L4, 23.2 ± 2.6; L5, 23.0 ± 1.3; L6, 21.3 ± 1.3<br>$Vglut1^{-/-};Vglut2^{fl/fl}$: L2/3, 29.8 ± 2.3; L4, 22.0 ± 3.6; L5, 20.5 ± 1.9; L6, 24.0 ± 3.1	[brains] $Vglut1^{+/+};Vglut2^{+/+}$, n = 4; $Vglut1^{-/-};Vglut2^{fl/fl}$, n = 4	Two-way ANOVA with Tukey-Kramer HSD test	L2/3, *p* = 0.82; L4, *p* = 0.99; L5, *p* = 0.85; L6, *p* = 0.75
		M1 (mean ± s.d.)	$Vglut1^{+/+};Vglut2^{+/+}$: L2/3, 24.6 ± 4.0; L4, 13.6 ± 2.4; L5, 30.1 ± 2.4; L6, 26.8 ± 4.2<br>$Vglut1^{-/-};Vglut2^{fl/fl}$: L2/3, 20.2 ± 2.4; L4, 11.5 ± 1.8; L5, 35.4 ± 2.3; L6, 28.5 ± 1.3	[brains] $Vglut1^{+/+};Vglut2^{+/+}$, n = 4; $Vglut1^{-/-};Vglut2^{fl/fl}$, n = 4	Two-way ANOVA with Tukey-Kramer HSD test	L2/3, *p* = 0.36; L4, *p* = 0.95; L5 = 0.17; L6, *p* = 0.98
Figure S4E	PV cell density (cells/mm^2) in S1		$Vglut1^{+/+};Vglut2^{+/+}$: mean = 201.1, median = 211.4, inter-quartile range = 28.0<br>$Vglut1^{-/-};Vglut2^{fl/fl}$: mean = 114.6, median = 113.8, inter-quartile range = 6.9<br>% change = -43.0%	[brains] $Vglut1^{+/+};Vglut2^{+/+}$, n = 4; $Vglut1^{-/-};Vglut2^{fl/fl}$, n = 4	Unpaired *t*-test	*p* < 0.01 (**)
Figure S4E	PV cell density (cells/mm^2) in M1		$Vglut1^{+/+};Vglut2^{+/+}$: mean = 198.9, median = 204.6, inter-quartile range = 18.6<br>$Vglut1^{-/-};Vglut2^{fl/fl}$: mean = 75.4, median = 79.6, inter-quartile range = 18.2<br>% change = -62.0%	[brains] $Vglut1^{+/+};Vglut2^{+/+}$, n = 4; $Vglut1^{-/-};Vglut2^{fl/fl}$, n = 4	Unpaired *t*-test	*p* < 0.001 (***)
**FIGURE S5**	**Measurement**		**Values**	**N**	**Statistical test**	**p-value**
Figure S5C	PV+ mCherry+ percentage (mean ± s.d.)		4.0 ± 0.8%	[brains] *Nkx2-1-Cre*, n = 3		
	ChAT+ mCherry+ percentage (mean ± s.d.)		2.9 ± 1.5%	[brains] *Nkx2-1-Cre*, n = 3		
